# Seven mitochondrial genomes of tribe Hylurgini (Coleoptera: Curculionidae: Scolytinae) in Eurasia and their phylogenetic analysis

**DOI:** 10.1371/journal.pone.0313448

**Published:** 2024-11-05

**Authors:** Na An, Yuan Yuan, Sixun Ge, Xudong Zhang, Lili Ren, Alain Roques, Youqing Luo

**Affiliations:** 1 Beijing Key Laboratory for Forest Pest Control, College of Forestry, Beijing Forestry University, Beijing, China; 2 IFOPE, Sino-French Joint Laboratory for Invasive Forest Pests in Eurasia, INRAE URZF and Beijing Forestry University, Beijing, China; 3 INRAE URZF, Orléans, France; Bayero University Kano, NIGERIA

## Abstract

The Hylurgini tribe (Coleoptera: Curculionidae: Scolytinae) comprises commercially significant bark beetles, including invasive species within the genera *Dendroctonus* and *Hylurgus*. These invasive species coexist with native *Tomicus* species of Hylurgini and cooperatively infest host trees in China. However, we lack sufficient mitochondrial genome data of Hylurgini to conduct phylogenetic studies, clarify the phylogenetic relationships of the above species, and improve the understanding of niche divergence and common hazards. Here, we sequenced and analyzed the mitochondrial genomes of seven Hylurgini species, including *Dendroctonus valens*, *Hylurgus ligniperda*, *Hylurgus micklitzi*, *Tomicus piniperda*, *Tomicus brevipilosus*, *Tomicus minor* and *Tomicus yunnanensis*. All sequenced mitochondrial genomes ranged from 15,339 bp to 17,545 bp in length, and their AT contents ranged from 73.24% to 78.81%. The structure of the seven mitochondrial genomes was consistent with that of ancestral insects. Based on 13 protein-coding genes from the reported mitochondrial genomes of 29 species of bark beetles, we constructed phylogenetic trees using maximum likelihood and Bayesian inference methods. The topology of the two phylogenetic trees was almost consistent. The findings elucidated the taxonomy classification of Hylurgini and the evolutionary connections of its sister taxa within the Scolytinae. This study offers insights for examining the evolutionary connections between invasive and native bark beetles, as well as the molecular identification and detection of newly invading species.

## 1. Background

Bark and ambrosia beetles comprise the subfamilies Scolytinae and Platypodinae in the family Curculionidae [1–7]. Hylurgini (Coleoptera: Curculionidae: Scolytinae), is a large group of Scolytinae which contains 14 genera: *Chaetoptelius* Fuchs, *Dendroctonus* Erichson, *Dendrotrupes* Broun, *Hylurdrectonus* Schedl, *Hylurgonotus* Schedl, *Hylurgopinus* Swaine, *Hylurgus* Latreille, *Pachycotes* Sharp, *Pseudohylesinus* Swaine, *Pseudoxylechinus* Wood & Huang, *Sinophloeus* Brèthes, *Tomicus* Latreille, *Xylechinosomus* Schedl, and *Xylechinus* Chapuis [4, 5, 8–12]. There are three genera in the tribe Hylurgini occurring in China, *i*.*e*., *Tomicus*, *Dendroctonus* and *Hylurgus*.

Many investigations have identified two or more species within the tribe Hylurgini that cooperatively damage their hosts in the same location. In some areas, the simultaneous species of Hylurgini are all native and belong to the same genus. For example, in 1997, a survey was conducted in Kunming City and Shuangbai County, Chuxiong City, Yunnan Province. *Tomicus minor* (Hartig) and *T*. *yunnanensis* concurrently inflicted damage on *Pinus yunnanensis*, leading to tree vulnerability and expedited mortality [13–15]. In 2011, *Tomicus*. *brevipilosus*, *T*. *minor* and *T*. *yunnanensis* were reported to co-occur in Qujing County, Jianshui City and Chuxiong City of Yunnan Province [16, 17]. In Arizona, USA, *Dendroctonus frontalis* Zimm. and *Dendroctonus brevicomis* LeConte were found on the same tree [18, 19]. In some areas, the invasive species of Hylurgini became co-damaging with indigenous species. *T*. *piniperda* and *H*. *ligniperda* were found to damage the host together in the Muping District of Yantai City, Shandong Province. This phenomenon was also observed in *Dendroctonus valens* and *T*. *piniperda* at Heilihe National Nature Reserve, Inner Mongolia.

There are many important forest pests in the tribe Hylurgini. Some species could cause massive damage during outbreaks. For example, *Dendroctonus ponderosae* Hopkin, a native species of western North America, caused massive forestry damage to more than 1.2 million hectares in 2004 [20]. *D*. *valens* and *H*. *ligniperda*, have been introduced from their indigenous habitats and have become significant invasive species, both caused significant destruction to forests following their introduction to China [7, 21–23]. Since its initial identification in 2020, *H*. *ligniperda* has inflicted significant damage on *Pinus thunbergi*i inside the coastal shelter belt of Moping District, Yantai City, Shandong Province. [24]. The invasive *D*. *valens* was introduced into China in 1998, mainly damaging healthy *Pinus tabuliformis* with a diameter at breast height greater than 10 cm or more than 30 years old [25, 26]. *Tomicus* spp., found in many regions of China, damage pine trees by feeding on the shoot and trunk [27]. Since the 1980s, in Yunnan Province alone, the damaged area has reached more than 10 × 10^4^ hm^2^ all year round [28].

In the study by Johnson *et al*. [29], 251 protein-coding genes from the subfamily Scolytinae were analyzed to establish the phylogenetic relationships of the subfamily Scolytinae, which consists of 22 tribes, including two species of *Dendroctonus* in the tribe Hylurgini. However, in morphological classification, the tribes of Xyleborini, Corthylini and Cryphalini are monophyletic [29–32]. According to the findings of Johnson *et al*., Xyleborini is associated with Dryocoetini, Corthylini is polyphyletic, and Cryphalini is significantly polyphyletic [29]. The phylogenetic relationships of this subfamily are still confusing due to the conflicting phylogenetic frameworks mentioned above, and data on Hylurgini are lacking, so further clarification is needed.

At present, mitochondrial genomes have been used in a wide range of studies such as species identification, molecular evolution and molecular markers for comparative genomics, and are of great significance in population genetics and evolution [26, 33–39]. Insect mitochondrial genome is more easily amplified than the nuclear genome, lacking non-coding regions (i.e., introns), maternal inheritance, low recombination rate, small size, rapid mutation rate, and so on [40–42]. Currently, the mitochondrial genomes of Coleoptera have been released in GenBank with 572 species, and 39 species of Scolytinae have complete mitochondrial genome data [43, 44]. Three species of Hylurgini have complete mitochondrial genomes, including *D*. *valens*, *D*. *rufipennis* and *H*. *ligniperda* [45]. The studies on Hylurgini were mainly focused on morphology and molecular identification based on the cytochrome c oxidase subunit I (*COI*) gene [46]. However, morphological characteristics are ineffective in solving intergeneric and interspecific phylogenetic relationships [16, 47–50]. The mitochondrial gene data of Hylurgini in GeneBank was seriously lacking except for the cytochrome oxidase I (*COI*) gene, which hindered the development of phylogenetic studies on it.

Here, we sequenced, assembled, and annotated the mitochondrial genomes of seven Hylurgini species, including *D*. *valens*, *H*. *ligniperda*, *H*. *micklitzi*, *T*. *piniperda*, *T*. *brevipilosus*, *T*. *minor*, *T*. *yunnanensis* and *H*. *micklitzi*. They have caused severe damage in China except for *H*. *micklitzi*. *H*. *micklitzi* currently does not distribute in China, but has extensive distribution in Europe. The mitochondrial genomes of seven Hylurgini species were compared and analyzed to understand their structure and base composition and to clarify the phylogenetic relationship between the tribe Hylurgini and other sister groups in Scolytinae. Phylogenetic studies of the tribe Hylurgini can provide us with a possible evolutionary framework to clarify the phylogenetic relationships of these species. Understanding phylogenetic relationships can also further advance knowledge of their niche changes and common hazards. Moreover, the members of the tribe Hylurgini are generally small in size, with close morphology of sympatric species. Its identification relies on taxonomists and strict identification conditions. Sequencing the mitochondrial genome could also advance the development of molecular markers for the tribe Hylurgini. Our results have important implications for studying mitochondrial genomes and clarifying the phylogenetic relationship.

## 2. Materials and methods

### 2.1 Sampling and genomic DNA extraction

**Fig 1:**
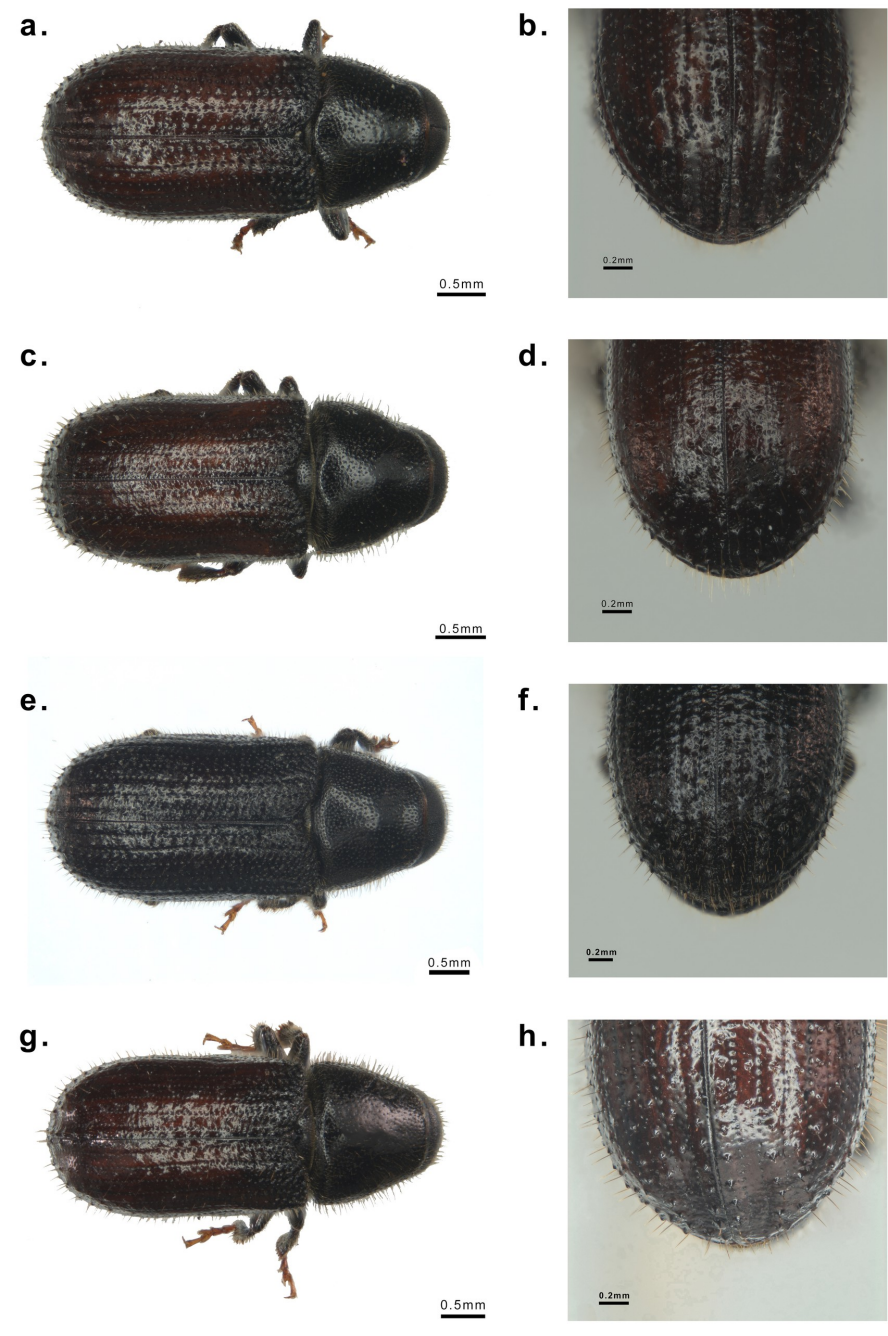
Morphological characteristics of Hylurgini. a. Adult dorsal view of *Tomicus brevipilosus*, b. Eytral declivity of *Tomicus brevipilosus*, c. Adult dorsal view of *Tomicus minor*, d. Eytral declivity of *Tomicus minor*, e. Adult dorsal view of *Tomicus piniperda*, f. Eytral declivity of *Tomicus piniperda*, g. Adult dorsal view of *Tomicus yunnanensis*. h. Eytral declivity of *Tomicus yunnanensis*.

**Fig 2:**
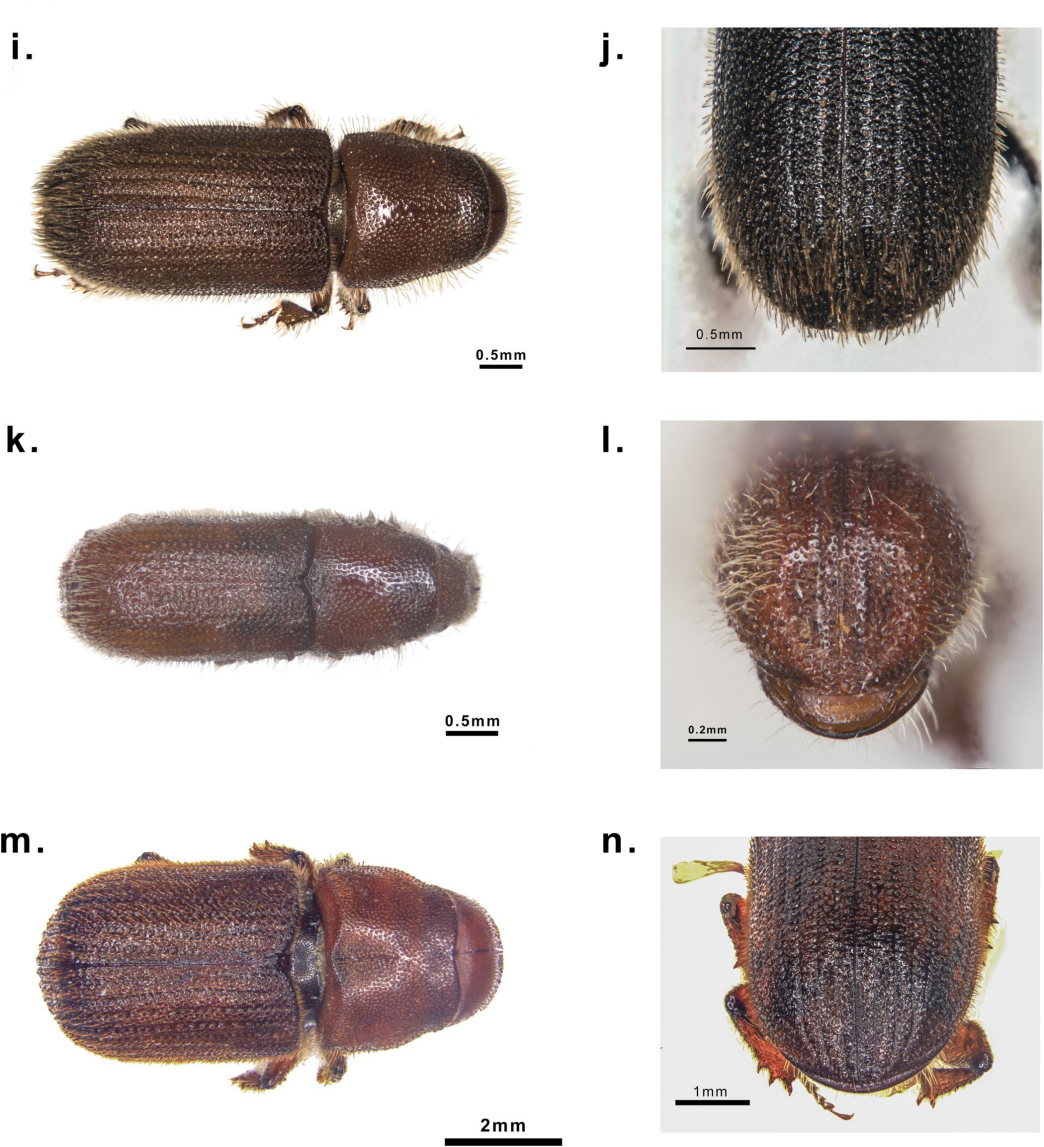
Morphological characteristics of Hylurgini. i. Adult dorsal view of *Hylurgus ligniperda*, j. Eytral declivity of *Hylurgus ligniperda*, k. Adult dorsal view of *Hylurgus micklitzi*, l. Eytral declivity of *Hylurgus micklitzi*, m. Adult dorsal view of *Dendroctonus valens*, n. Eytral declivity of *Dendroctonus valens*.

Samples of seven species of bark beetle adults were collected from China and France. From October 16^th^ to 23^th^, 2021. More than 600 individuals of *T*. *yunnanensis*, *T*. *minor*, *T*. *brevipilosus* were collected from three sites in Yunnan Province. *D*. *valens* was collected in Heilihe National Nature Reserve, Inner Mongolia. *H*. *ligniperda* and *T*. *piniperda* were collected from Muping, Shandong Province. *H*. *micklitzi* has been collected by the Orléans Forest Zoology Research unit (French Institute for Agri-Food and Environmental Research- INRAE), at Le Thoronet, Southern France by attractive traps on July 15^th^ 2021 (Table 1). The above specimens were stored in the laboratory (College of Forestry, Beijing Forestry University) refrigerator in absolute ethyl alcohol at −20°C. A Zeiss AxioZoom V16 type microscope was used to observe and take photos of the samples (Figs 1 and 2). We identified the specimens with reference to these previous studies [9, 16, 49, 51]. According to the instructions, the AxyPrep Multisource Genomic DNA Miniprep Kit (Axygen, USA) was used to extract the genomic DNA from the entire body of specimens from the same locality of each Hylurgini species. The total DNA quality was examined with 1% agarose gel, and the concentration was measured with a NanoDrop 2000 spectrophotometer (Thermo Scientific, USA).

**Table 1:** Collection information of Hylurgini species in this study.

Name	Location	Country	Longitude	Latitude	Accession number
*Dendroctonus valens* LeConte	Heilihe National Nature Reserve, Inner Mongolia	China	118.466830	41.407369	OP651189
*Hylurgus ligniperda* (Fabricius)	coastal shelterbelt in Moping District, Yantai City, Shandong Province	China	121.851217	37.457241	OP651193
*Hylurgus micklitzi* Wachtl	Le Thoronet	France	6.303889	43.451944	OP651194
*Tomicus brevipilosus* (Eggers)	Ninger Hani and Yi Autonomous County, Pu ’er City, Yunnan Province	China	101.238255	22.964431	OP651191
*Tomicus minor* (Hartig)	Pupeng Town, Xiangyun County, Dali Bai Autonomous Prefecture, Yunnan Province	China	100.915525	25.315671	OP644291
*Tomicus piniperda* (Linnaeus)	coastal shelterbelt in Moping District, Yantai City, Shandong Province	China	121.851217	37.457241	OP651192
*Tomicus yunnanensis* Kirkendall & Faccoli	Anaconda Pit, Panlong District, Kunming City, Yunnan Province	China	102.882109	25.202455	OP651190

### 2.2 Mitochondrial genome sequencing and assembly

Samples with qualified mass and concentration greater than 2 ng/μl were sent to Shanghai Majorbio Bio-pharm Technology Co., Ltd for high-throughput sequencing. The library was constructed and sequenced using the Illumina HiSeq X platform based on Sequencing by Synthesis (SBS) technique. Filter the raw data using *Hylastes attenuatus* (KX035212.1) as the reference sequence. Clean reads were assembled with the software GetOrganelle (https://github.com/Kinggerm/GetOrganelle) [52] under the K-mer = 21, 45, 65, 85, 105. To ensure the accuracy of the results, NOVOPlasty4.2 (https://github.com/ndierckx/NOVOPlasty) [53] was also used with *Hylastes attenuatus* (KX035212.1) as a seed sequence for mitochondrial genome assembly under K-mer = 33. Then the results of the two software were compared, and the full length of mitochondrial genome sequences of the seven species were obtained.

### 2.3. Mitochondrial genome annotation and analysis

MITOS (http://mitos2.bioinf.uni-leipzig.de/index.py) [54] was used to annotate the mitochondrial genome results of seven species. The type of reference gene set was Metazoa, and the Genetic Code was Invertebrate. The BLAST function of NCBI was used to confirm the accuracy of the annotation results. Based on the available mitochondrial genome data of the family Scolytinae, the start codons and stop codons with problems in 13 PCGs were manually adjusted. ARWEN (www.acgt.se/online.html) [55] and tRNAscan—SE 2.0 (http://trna.ucsc.edu/tRNAscan-SE/) were used to predict the secondary structure of tRNA and annotate the undiscovered tRNA [56]. Using the Tandem Repeats the Finder online server (http://tandem.bu.edu/trf/trf.html) to find concatenation repeats sequences [57]. The two rRNA genes were compared with *Dendroctonus rufipennis*, *Hylastes attenuates* Erichson and *Polygraphus poligraphus* (Linnaeus) of the subfamily Scolytinae, and manually adjusted by them. MEGA11 [58] was used to analyse the nucleotide codon usage frequency (RSCU) and nucleic acid composition. According to the formula, the AT-skew = [(A − T)/(A + T)] and GC-skew = [(G − C)/(G + C)] calculating nucleotide differences (AT deflection and GC deflection). The synonymous substitution rate (dS) and non-synonymous substitution rate (dN) were calculated by PALM 4.9j [59]. The heatmap was plotted using TBtools (Toolbox for biologists) [60]. Finally, CGView Server (http://stothard.afns.ualberta.ca/cgview_server) [61] was used to draw the mitochondrial genome maps.

### 2.4 Sequence alignment and phylogenetic analysis

Complete published mitochondrial genome sequences of 29 species from 10 tribes of Scolytinae were downloaded from the GenBank (S1 Table), and 13 protein-coding genes (PCGs) were extracted using the Phylosuite1.2.2 [62] software. MAFFT [63] was used to conduct multiple sequence alignment. Then, the sequences were concatenated to obtain a nucleotide sequence data set of protein-coding genes. The sequence of *Acrida cinerea* (NC_014887.1) was selected as an outgroup. We used IQ-TREE (maximum likelihood, ML) [64] and MrBayes 3.2.6 (Bayesian inference, BI) [65] of Phylosuite1.2.2 software to construct phylogenetic trees. In IQ-TREE, we selected the “Auto” model. The confidence level of the branch node was estimated using the 1,000 ultrafast bootstrap replicates. With the help of ModelFinder [66], the optimal nucleotide substitution model was selected as GTR + I + G + F, and MrBayes performed Bayesian inference under the GTR + I + G + F model, operating for 2,000,000 generations with two simultaneous runs, sampling once every 1,000 generations, and the burn-in fraction was set to 0.25. The value of the average standard deviation of split frequencies was less than 0.01, and there was little difference between the results of the two runs, and the parameters converged [67]. The tree files were visualized by iTOL (Interactive Tree Of Life) (http://itol.embl.de) [68]. Finally, MEGA11 software was used to calculate the genetic distance of Hylurgini.

## 3. Results

### 3.1 Mitochondrial genome structure and base composition of seven Hylurgini species

**Fig 3:**
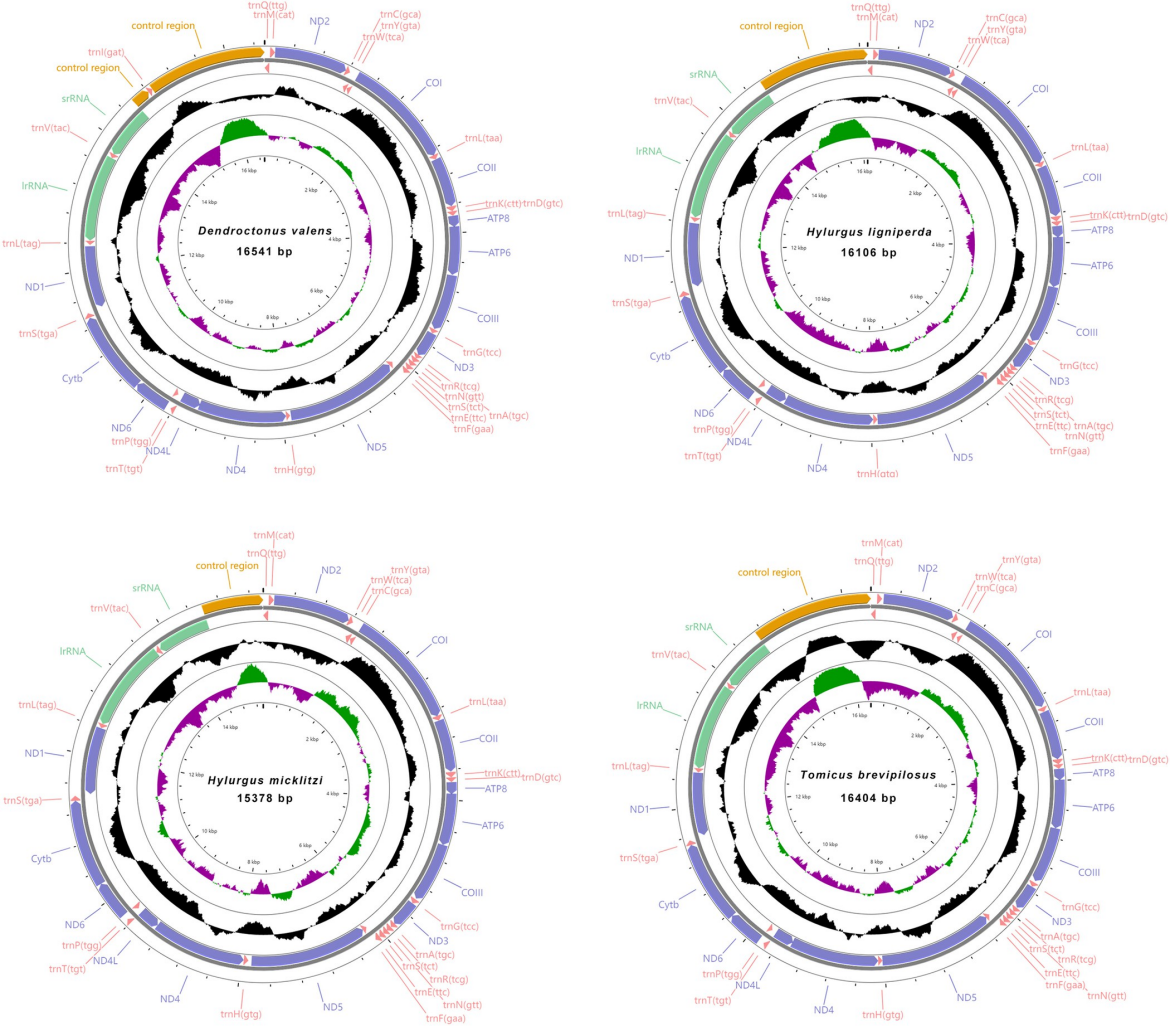
Mitochondrial genome circular maps of *Dendroctonus valens*, *Hylurgus ligniperda*, *Hylurgus micklitzi* and *Tomicus brevipilosus*.

**Fig 4:**
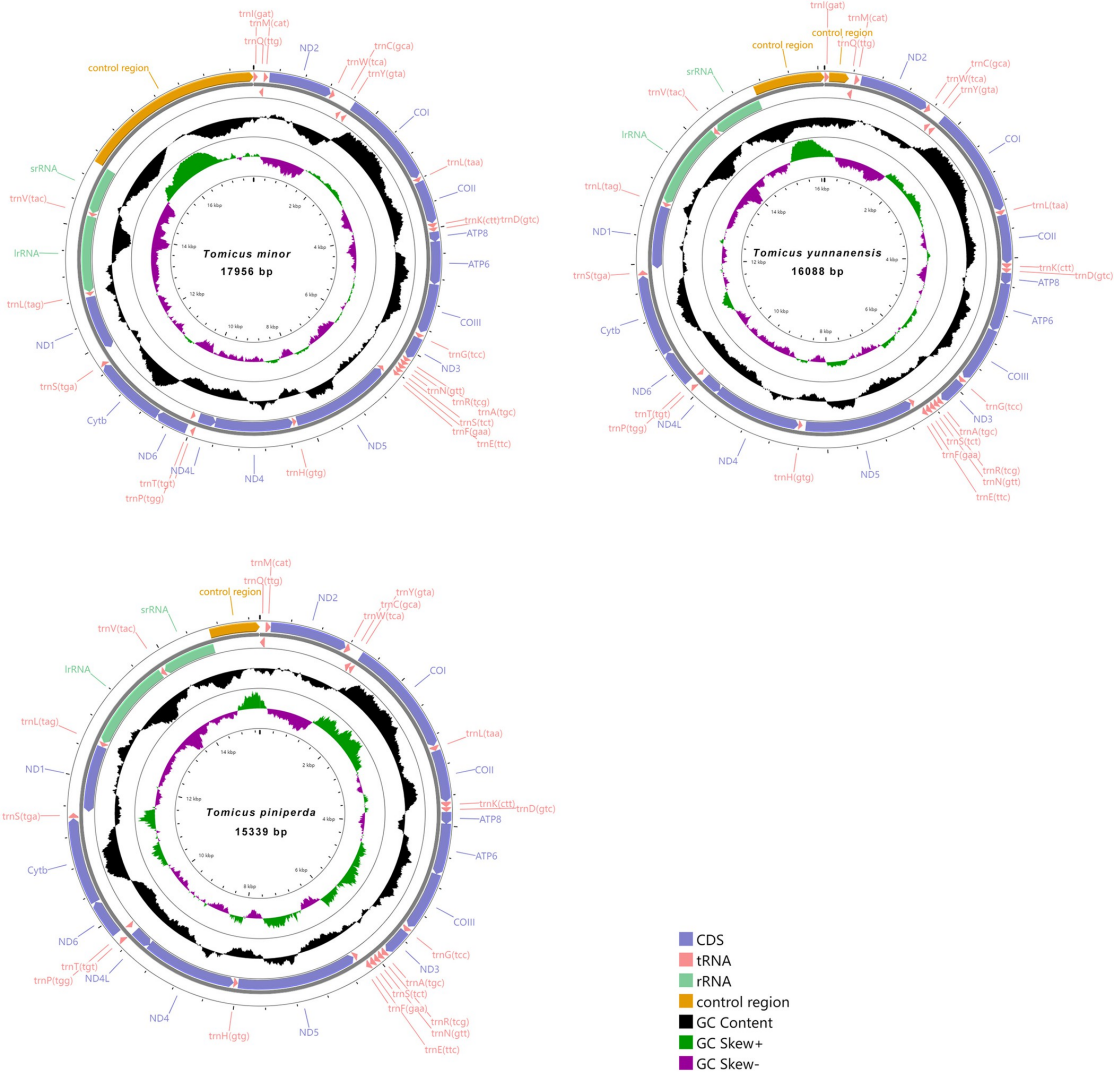
Mitochondrial genome circular maps of *Tomicus minor*, *Tomicus yunnanensis* and *Tomicus piniperda*.

The mitochondrial genome sequences of seven Hylurgini species ranged from 15,339 bp to 17,545 bp. The shortest was *T*. *piniperda* and the longest was *T*. *minor*. *T*. *minor*, *T*. *yunnanensis*, and *D*. *valens* were composed of a control region and 37 genes (13 protein-coding genes, 22 tRNA genes and two rRNA genes). The other four species, *T*. *brevipilosus*, *T*. *piniperda*, *H*. *ligniperda* and *H*. *micklitzi*, were missing *tRNA*^*Ile*^ genes, all of which were 36 genes (Figs 3 and 4). Among the seven newly sequenced mitochondrial genomes, nine protein-coding genes (*ND2*, *COI*, *COII*, *ATP8*, *ATP6*, *COIII*, *ND3*, *ND6*, *Cytb*), 14 tRNA genes (*tRNA*^*Ile*^, *tRNA*^*Met*^, *tRNA*^*Trp*^, *tRNA*^*Leu(UUR)*^, *tRNA*^*Lys*^, *tRNA*^*Asp*^, *tRNA*^*Gly*^, *tRNA*^*Ala*^, *tRNA*^*Arg*^, *tRNA*^*Asn*^, *tRNA*^*Ser(AGN)*^, *tRNA*^*Glu*^, *tRNA*^*Thr*^, *tRNA*^*Ser(UCN)*^) were distributed on the J strand (majority strand, encodes more genes in the DNA double strand), while the remaining 14 genes were on the N strand (minority strand, encodes a relatively small number of genes in the DNA double strand) [69, 70].

There were 13 non-coding regions in *D*. *valens*, 13 in *H*. *ligniperda* and *H*. *micklitzi*, 16 in *T*. *minor*, *T*. *brevipilosus* and *T*. *piniperda*, and 17 in *T*. *yunnanensis* at most. Except for the control region, the most extended non-coding region was in *tRNA*^*Ile*^*-tRNA*^*Gln*^ of *T*. *yunnanensis*, with a length of 281 bp. The overlap of the seven Hylurgini species varied from 8 to 14 places. Two overlapping regions, *tRNA*^*Lys*^-*tRNA*^*Asp*^ and *ATP8*-*ATP6*, were found in all seven bark beetle species.

The content of A+T in the whole mitochondrial genome of the seven Hylurgini species ranged from 73.24% to 78.81%, with the lowest content of *D*. *valens* and the highest content of *H*. *micklitzi*, showing an obvious AT bias. The AT-skew of the seven species was 0.024 to 0.080, and the GC-skew was negative, ranging from −0.239 to −0.158.

### 3.2 Mitochondrial protein-coding genes and relative synonymous codon usage

In seven mitochondrial genomes, the total length of 13 protein-coding genes ranged from 11,082 bp to 11,138 bp, accounting for 61.84% to 72.61% of the total sequence, in which *ND5* of *T*. *brevipilosus* was the longest, 1,723 bp. *ATP8* of *H*. *micklitzi*, *D*. *valens*, and *H*. *ligniperda* was the shortest, 156 bp. Most PCGs were started by the insect standard start codon ATN. However, the start codon of *T*. *minor*’s *COI* gene was TCG; the start codon of the protein-coding gene *ND1* of *T*. *yunnanensis*, *T*. *brevipilosus*, *H*. *micklitzi* and *T*. *piniperda* was TTG respectively. As for stop codon, five PCGs (*ND2*, *COI*, *ATP6*, *COIII*, *ND6*) ended with TAA in seven mitochondrial genomes sequenced in this study. *Cytb* of *T*. *brevipilosus* and *H*. *micklitzi*, *ND4L* of *H*. *ligniperda*, *ND1* of *T*. *yunnanensis* and *D*. *valens*, *ND3* of *H*. *micklitzi*, *D*. *valens* and *T*. *minor* all had TAG as the stop codon. *ND5* of *H*. *ligniperda* was terminated by TA residue. In contrast, the remaining PCGs including *COII*, *ND5*, *ND4* and *ND1* appeared to terminate with a T residue in seven species. For example, *ND5*, *ND4* and *ND1* of *H*. *micklitzi*, *ND5*, *ND4* of *D*. *valens*.

The AT content of PCGs was 71.6% to 77.9%. Contents of the third codon position AT *H*. *micklitzi*, *H*. *ligniperda*, *T*. *minor*, *T*. *yunnanensis* and *T*. *piniperda* (77.4% to 81.8%) were higher than those of the first (70% to 77.5%) and the second (69.5% to 78.3%) codon positions. In contrast, *D*. *valens* and *T*. *brevipilosus* had higher AT content at the first codon position (73% and 79.2%) than at the second (71.3% and 72.3%) and third (70.6% and 77.2%) codon positions. The AT-skew range of PCGs for the seven species was −0.152 to −0.129, and the range of GC-skew was −0.067 to −0.008.

**Fig 5:**
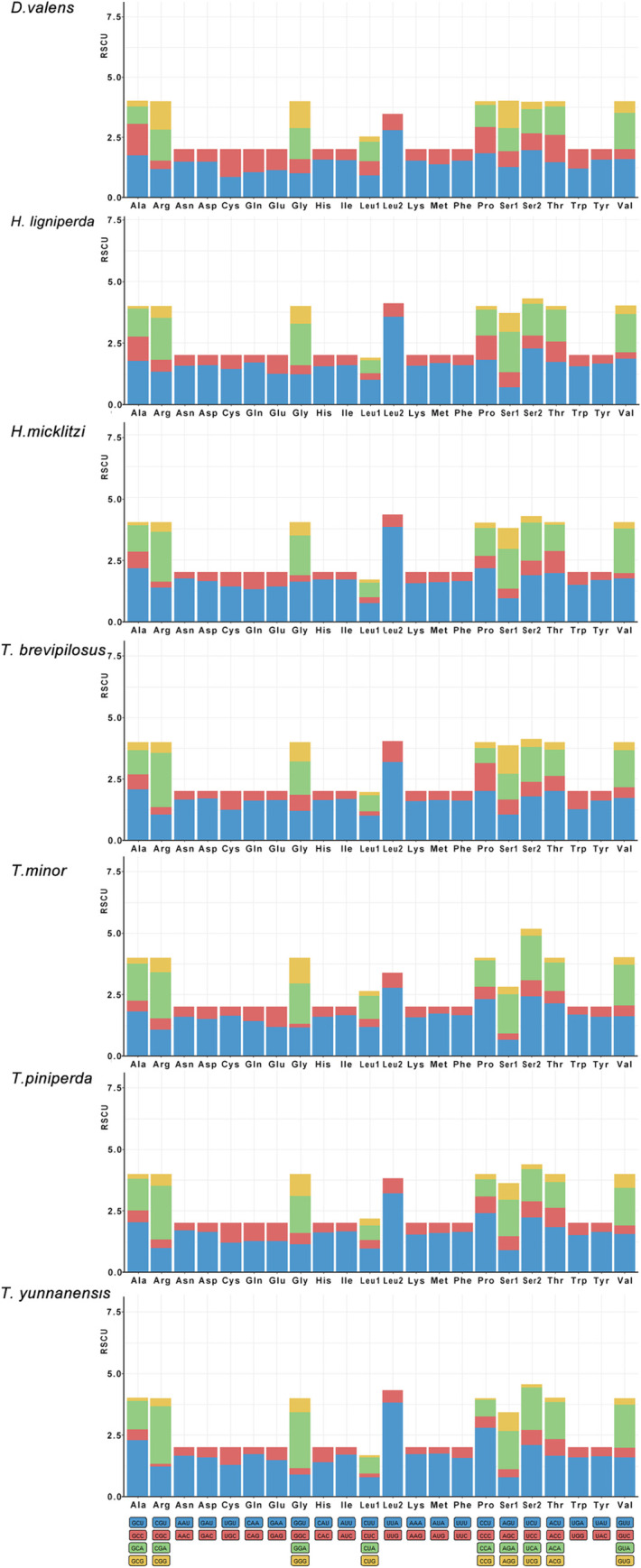
The relative synonymous codon usage (RSCU) in the mitochondrial genomes of seven Hylurgini species.

In addition, the relative synonymous codon usage (RSCU) analysis results showed that in the mitochondrial genome PCGs of seven Hylurgini species, there were 22 codon families containing 62 codons (excluding two stop codons). The codon families encoding amino acids Ala, Arg, Gly, Pro, Ser2, Thr, and Val are frequently used in all seven mitochondrial genomes (Fig 5). Among the seven mitochondrial genomes, UUU (Phe), UUA (Leu), CCU (Pro), GUU (Val), GCU (Ala), CGA (Arg), ACU (Thr), GGA (Gly) were the most frequently used. The five most frequent codons make up 10.94% to 15.16% of all PCG codons. The least frequently used codon was the CCG (Pro) codon of *T*. *yunnanensis*, with an RSCU of 0.07.

Compared with other synonymous codons, A or T ranked third in the double and quadruple degenerate synonymous codons were overused. In this study, there was a preference for codons, except CCU (Pro) (1.14), AGG (Ser1) (1.15), and GGG (Gly) (1.06), all of which ended in A or T (U). This suggests that, in addition to the effect of selection on the bias of codon usage, the mutation pressure at the third codon position from GC to AT is higher than in the opposite direction (AT to GC), as well as at the first and second codon positions [71]. In addition, the five most frequently used codons in each of the seven mitochondrial genomes were not identical. For example, the five codons with the highest frequency (maximum RSCU value) of *D*. *valens* and *H*. *ligniperda* were UUA(Leu), CCU(Pro), UCU(Ser2), GUU(Val), and GCU(Ala), that of *H*. *micklitzi* and *T*. *brevipilosus* were UUA (Leu), CCU (Pro), CGA (Arg), ACU (Thr) and GCU (Ala), and that of *T*. *minor* were UUA (Leu), CCU (Pro), UCU (Ser2), CGA (Arg) and ACU(Thr). It could be seen that different species of the same family may have different preferences for using synonymous codons. However, the specific situation needs further analysis.

### 3.3 Synonymous substitution rate and the non-synonymous substitution rate of mitochondrial protein-coding genes

**Fig 6:**
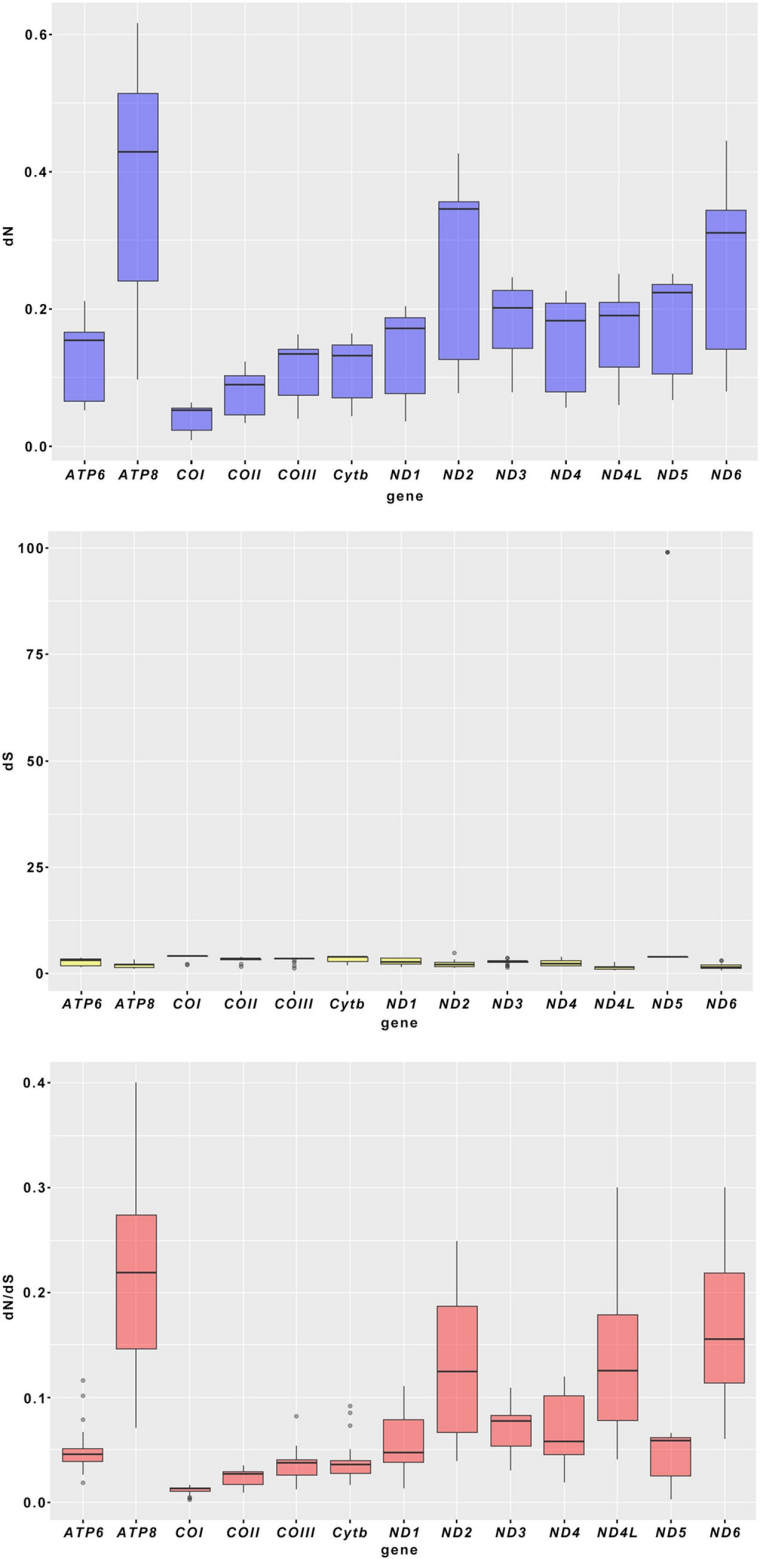
The dN, dS, and dN/dS values of 13 PCGs in the mitochondrial genomes of Hylurgini species.

**Fig 7:**
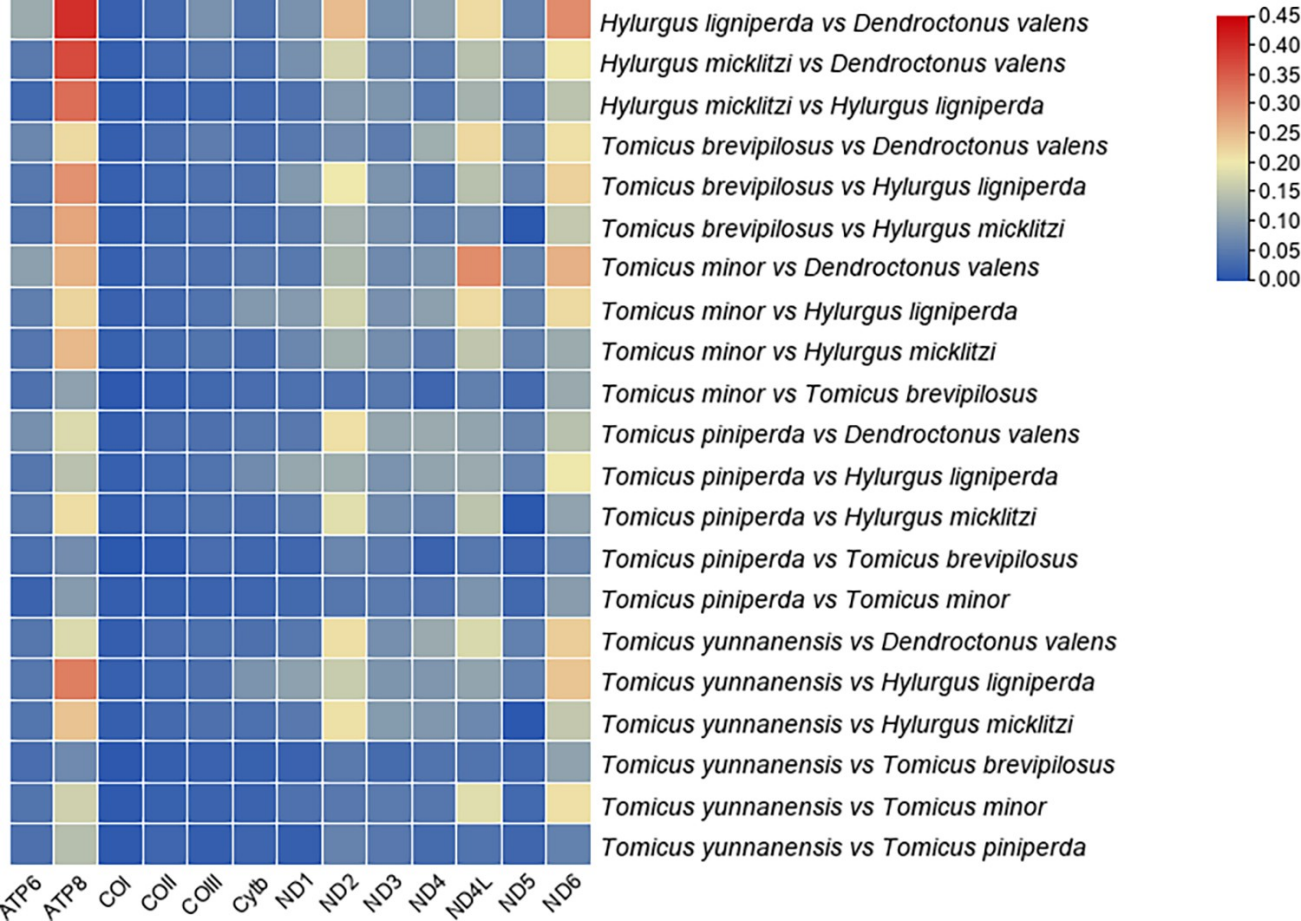
The HeatMap of 13 PCGs in the mitochondrial genomes of Hylurgini species.

To determine the molecular sequence evolution mechanism of Hylurgini, we also analysed the Synonymous substitution rate (dS) and the Non-synonymous substitution rate (dN) of each PCGs (Fig 6). The results showed that all PCGs of seven mitochondrial genomes evolved under purification selection (dN/dS < 1). On the one hand, the dN/dS values of all PCGs were very low, ranging from 0.0021 to 0.4001, indicating strong purification selection. The *ATP8* (dN/dS = 0.3703) of *H*. *micklitzi* vs. *D*. *valens* and the *ATP8* (dN/dS = 0.4001) of *H*. *ligniperda* vs. *D*. *valens* were closer to 1, indicating that the purification selection of this gene was looser. In the Hylurgini, the purified selected genes were sorted by decreasing selection intensity (increasing dN/dS value) as follows: *COI* > *COII* > *Cytb*> *COIII* > *ATP6* > *ND5* > *ND1* > *ND3* > *ND4* > *ND4L* > *ND2* > *ND6* > *ATP8*. None of the PCGs had dN/dS > 1, indicating that Hylurgini PCGs were mainly influenced by purification selection in the evolutionary process, while *ATP8* encountered relatively small purification selection pressure. HeatMap shows that *ND2*, *ND4L* and *ND6* were under loose purification selection except for *ATP8* (Fig 7). While CO genes (*COI*, *COII* and *COIII*) were under relatively strong purification selection pressure. In conclusion, purification selection would eliminate harmful mutations in the population, and all PCGs of the Hylurgini can be used for our phylogenetic analysis.

### 3.4 Transfer and ribosomal RNA genes of mitogenome

**Fig 8:**
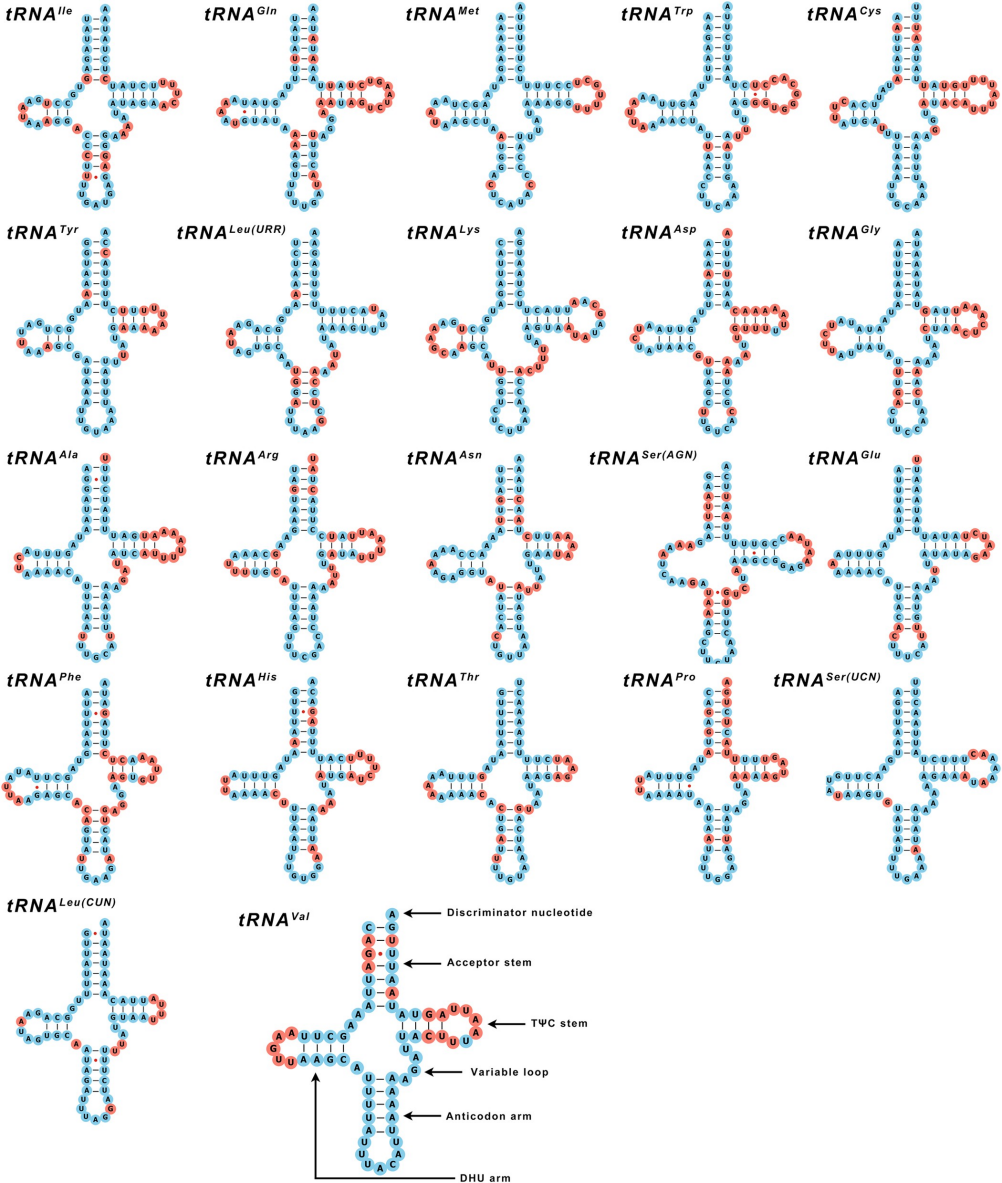
Secondary structure of 22 tRNA genes in the mitochondrial genome from seven species of Hylurgini. Conserved sites and mutation sites were indicated in blue and red, respectively, and mismatched base pairs were marked with red solid dots.

A total of 22 tRNA genes were annotated by MITOS2. The length of tRNA genes ranged from 60 bp (*tRNA*^*Cys*^ of *D*. *valens*) to 71 bp *(tRNA*^*Lys*^ of *D*. *valens*, *H*. *micklitzi*, *T*. *minor*, *T*. *brevipilosus*, *T*. *yunnanensis* and *T*. *piniperda*). The average length of t-RNA is 64 bp. The secondary structure of 21 tRNA genes was typical of the cloverleaf, and only the *tRNA*^*SER(AGN)*^ was atypical in all seven Hylurgini, lacking a dihydrouracil (DHU) arm (Fig 8).

In addition, the secondary structure prediction results showed that unmatched base pairs occurred on the stem of some tRNA genes. The mismatched pairs of G-U occurred on 11 tRNA genes, a total of 84 pairs. *H*. *ligniperda* had the most mismatches and *T*. *brevipilosus* had the least. The G-U mismatched pairs of *tRNA*^*Ala*^, *tRNA*^*Phe*^, *tRNA*^*His*^, *tRNA*^*Leu(CUN)*^, and *tRNA*^*Val*^ occurred on acceptor stems. The mismatch pairs of *tRNA*^*Gln*^, *tRNA*^*Tyr*^, *tRNA*^*Phe*^ and *tRNA*^*Pro*^ occurred on DHU arm, *tRNA*^*Ile*^ on the anticodon arm, and *tRNA*^*Trp*^, *tRNA*^*Ser(AGN)*^ occurred on TΨC stem (Fig 8).

The two rRNA, *srRNA* and *lrRNA* genes, were on the N-strand. The length of the *lrRNA* ranged from 1,283 bp to 1,301 bp. The shortest was *H*. *micklitzi* and the longest was *T*. *yunnanensis*. The length of *srRNA* ranged from 765 bp to 782 bp. The shortest was *H*. *micklitzi* and the longest was *T*. *minor*. The AT content of *lrRNA* ranged from 80.6% to 82.5%, which was higher than that of *srRNA* (79.3% to 80.6%). All of them had high A + T preference.

### 3.5 Phylogenetic analysis

**Fig 9:**
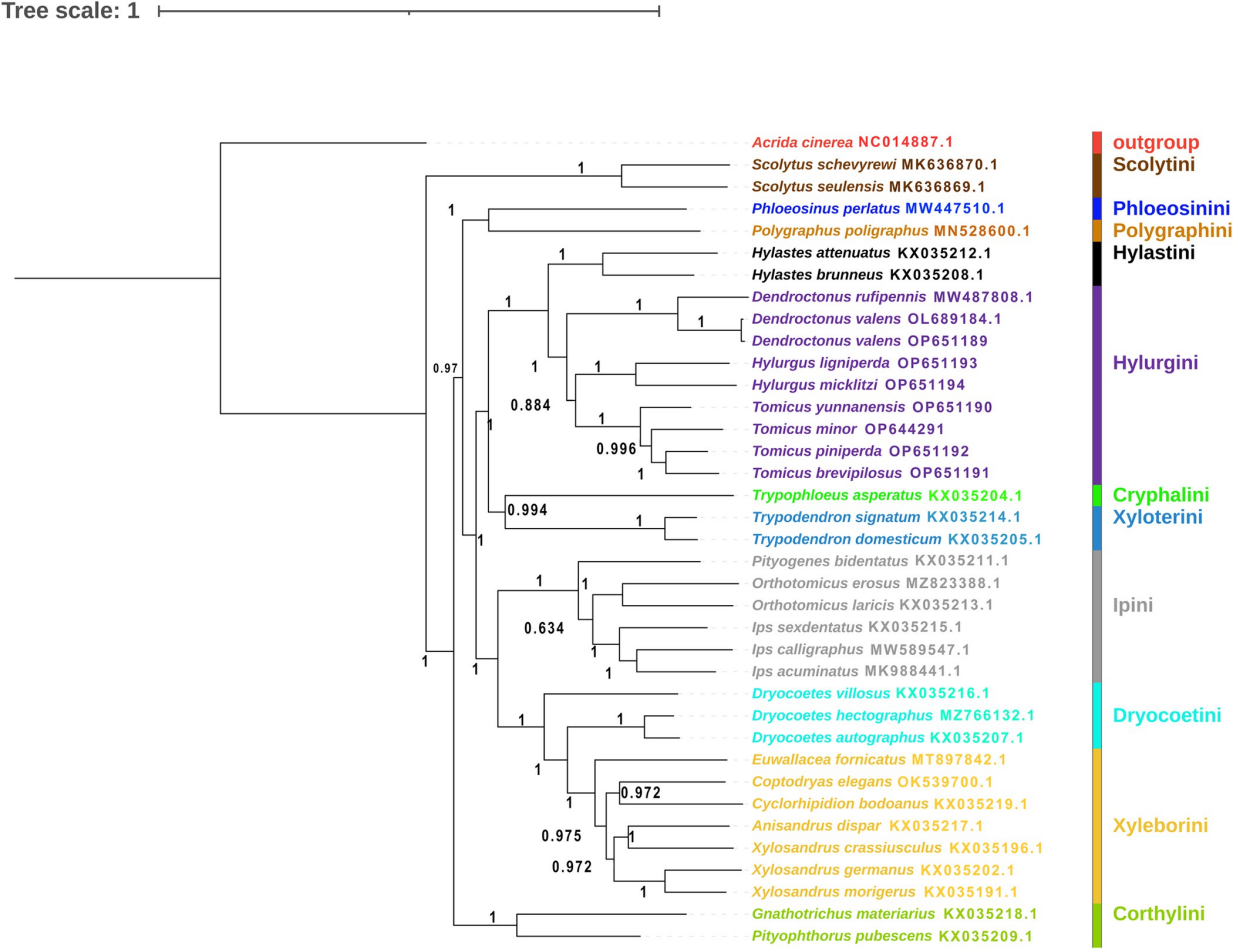
A phylogenetic tree constructed based on 13 PCGs of the mitochondrial genomes. The numbers at nodes indicate Bayesian posterior probabilities.

**Fig 10:**
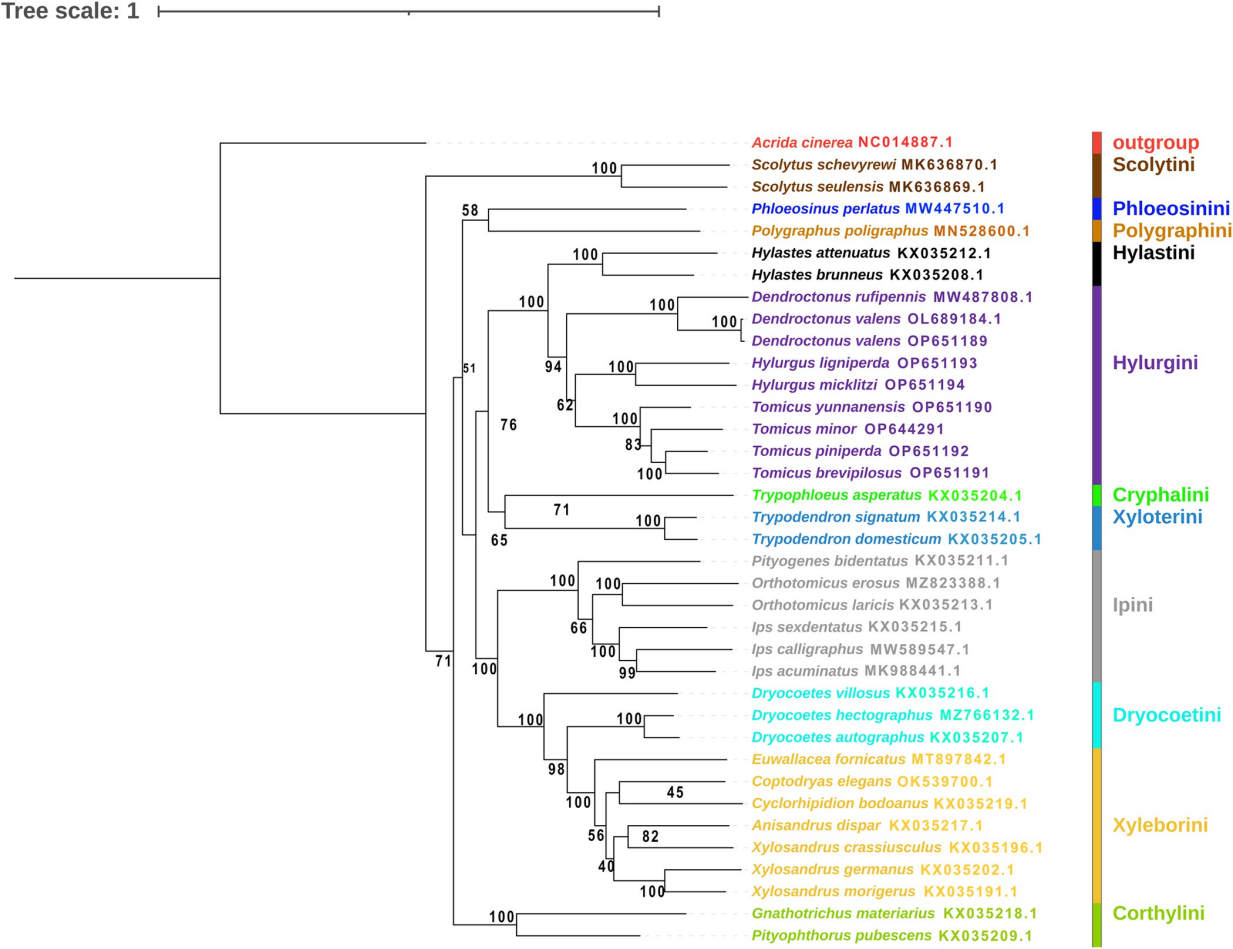
A phylogenetic tree constructed based on 13 PCGs of the mitochondrial genomes. The numbers at nodes indicate ML bootstrap support values.

We performed a phylogenetic analysis of 13 PCGs from 37 mitochondrial genomes (from 11 tribes of the subfamily Scolytinae). The same topologies were presented by constructing phylogenetic trees from two methods, likelihood (ML) and Bayesian inference (BI) (Figs 9 and 10). In the Bayesian inference, the tribe of Scolytini, Hylastini, Hylurgini, Xyloterini, Ipini, Xyleborini and Corthylini showed high bootstrap values. In the likelihood analysis, the tribe of Scolytini, Hylastini, Xyloterini, Ipini, Corthylini and Hylurgini showed high bootstrap values. These results allowed better support for the monophyly of the tribe and genera of our studied species. The results of the two methods showed consistent phylogenetic relationships. Scolytini was the first to separate from the other groups and clustered as a monophyletic branch. *Phloeosinus perlatus* of the tribe Phloeosinini and *Polygraphus poligraphus* of the tribe Polygraphini clustered as a monophyletic branch. The Hylastini and Hylurgini are monophyletic. Cryphalini and Xyloterini clustered into a monophyletic clade. Ipini with Dryocoetini, and Xyleborini formed a monophyletic lineage with Ipini sister to Dryocoetini and Xyleborini. *Gnathotrichus materiarius* and *Pityophthorus pubescens* of the tribe Corthylini formed a monophyletic branch. These results were consistent with morphological and single gene/multiple genes of the mitochondrial genomic PCGs tree [72].

## 4. Discussion

### 4.1 Mitochondrial genomes comparison between Hylurgini and the other tribes of Scolytinae

The seven mitochondrial genomes newly sequenced were shorter than those of their sister groups published to NCBI (S2 Table). The mitochondrial genome structure of the seven bark beetles, as determined through our assembly and annotation, was congruent with previously known species of the tribes Phloeosinini and Ipini [72]. The mitochondrial genome structure was identical to that of ancestry insects [33]. All 22 tRNA genes of *T*. *minor*, *T*. *yunnanensis* and *D*. *valens*, were successfully annotated by MITOS2. However, *tRNA*^*Ile*^ of *T*. *brevipilosus*, *T*. *piniperda*, *H*. *ligniperda* and *H*. *micklitzi* were not annotated. The proximity of *tRNA*^*Ile*^ to the regulatory region and its abundance of A and T bases restricts Illumina sequencing runs, resulting in the detection of only 36 genes without comprehensive sequencing. The content of A + T of Hylurgini ranged from 73.24% to 78.81%, the content of *D*. *valens* was the lowest, and *H*. *micklitzi* was the highest, showing an obvious AT bias. In general, the species of Hylurgini had a low content of A + T compared with other species of Scolytinae (S2 Table). The AT-skew of the whole mitochondrial genome of the seven species was positive, indicating that there were more bases A than C. The GC-skew was negative, indicating that the content of G in the mitochondrial genome of Hylurgini was less than C, which was consistent with the results of other published Scolytinae [72].

In the 13 PCGs of seven mitochondrial genomes, TCG and TTG were also used as the start codons in addition to the standard ATN start codon. The *COI* gene of *T*. *minor* was taken TCG as the start codon, while the mitochondrial genomes of other bark beetles have not been reported, but the *COI* gene of other insects, such as Diptera, has the same occurrence [73, 74]. TTG was the start codon of the *ND1* of *T*. *yunnanensis*, *T*. *brevipilosus*, *H*. *micklitzi* and *T*. *piniperda*, which also occurs on the *ND1* gene of the genus *Ips* [72]. This study also discovered stop codons, including TAG, T residues, and TA residues, in addition to TAA. TAG was the stop codon of *Cytb*, *ND4L*, *ND1* and *ND3*, which also occurred in the PCGs of the published species of the Scolytinae [72, 75]; *ND5* of *H*. *ligniperda* was terminated by TA residue. *COII*, *ND5*, *ND4* and *ND1* all are terminated with a T residue. This is also consistent with the mitochondrial genome of other published bark beetles [72].

The total AT content of the 13 PCGs ranged from 71.6% to 77.9%, higher than the average AT content of the 29 published mitochondrial genome PCGs we referred to: 70.37% (S2 Table). We also compared our nucleotide composition of the codon position of the concatenated PCGs with other published related species. The results of *H*. *micklitzi*, *H*. *ligniperda*, *T*. *minor*, *T*. *yunnanensis*, and *T*. *piniperda* were consistent with the published related species such as *Hylastes* and *Ips*. The content of the third codon positions was higher than that of the first and the second codon positions (S2 Table). The AT-skew and GC-skew values of PCGs of seven mitochondrial genomes were negative, indicating that the number of base T was greater than A. The number of base C was greater than G in PCGs, which was consistent with the published results of the tribe Ipini and Hylastini [72].

RSCU is the ratio between the frequency at which a codon is observed and the expected frequency at which the codon of any particular amino acid is equally used [76]. Thus, RSCU directly exhibits a using deviation between synonymous codon and uniform. RSCU = 1 indicates that the codon is randomly used (unbiased), RSCU > 1 suggests that the codon is used more frequently (biased), and RSCU < 1 indicates that the codon is used less frequently [77]. In the seven mitochondrial genomes, the number of synonymous codons with high using frequency was 26 (*T*. *yunnanensis* and *T*. *piniperda*), 28 (*H*. *micklitzi*, *T*. *brevipilosus* and *H*. *ligniperda*), and 30 (*T*. *minor*), 32 (*D*. *valens*), indicating a preference for these codons in their respective species. This indicates that species preferentially select optimal synonymous codons, as some chosen codons are more frequently found at conserved places [78].

Only 21 tRNA genes were detected in four of the seven sequenced mitochondrial genomes, and the missing gene was *tRNA*^*Ile*^. This phenomenon has also occurred in other species of Scolytinae [79]. The loss of *tRNA*^*Ile*^ is due to the sequencing method. The second-generation sequencing sometimes fails to detect the AT-rich area, resulting in the loss of the gene near the AT-rich area. In the insect mitochondrial genome, *tRNA*^*SER(AGN)*^ usually misses the DHU arm rather than the classic cloverleaf structure. This phenomenon has also been observed in the mitochondrial genomes of other species of Scolytinae, *e*.*g*., the genus of *Ips* [72]. According to the results, the base mismatch in the secondary structure of tRNA mainly occurred in the DHU arms, and little in the Anticodon arms. Consequently, we can infer that various regions of tRNA exhibit distinct conservative traits, with the Anticodon arm being the most conserved. In addition, most mutation sites were located on the ring, while fewer were on the arm, indicating that the stem was more conservative.

### 4.2 Phylogeny of Hylurgini

The phylogenetic relationships among some taxa of Scolytinae were still unclear. For example, the tribe Dryocoetini was shown to be paraphyletic, with *D*. *villosusu* being sister to the other *Dryocoetes* species and the tribe Xyleborini. Among Hylurgini, our results showed that all three genera exhibit good monophyly. In the genus *Tomicus*, *T*. *brevipilosus*, *T*. *piniperda* and *T*. *minor* clustered into a monophyletic branch, which sister to *T*. *yunnanensis*. The *T*. *brevipilosus* and *T*. *piniperda* clustered into a monophyletic branch, separated from *T*. *minor*.

Hylurgini was sister to the tribe Hylastini in our phylogenetic analysis, aligning closely with the classification based on morphological characteristics [9]. The position of *Dryocoetes villosus* was undecided. Jordal [80] and Pistone [81] also indicated that the genus *Dryocoetes* was not monophyletic by molecular phylogeny and constitutes at least two sister groups, which requires a revision of it.

The genetic distance data of 37 species of beetles calculated by Mega 11 are shown in S3 Table. *T*. *piniperda* is comparatively more distantly related to *H*. *ligniperda* in the *Hylurgus* genus. *H*. *ligniperda* is more closely related to *T*. *yunnanensis*, a member of the *Tomicus* genus. *T*. *minor* is the most distantly related to *T*. *yunnanensis* and is distantly related to three other beetle species in the same genus. The results were not consistent with the phylogenetic results. In the phylogenetic results, *T*. *yunnanensis* was the most distant from the other three species of the same genus. *T*. *brevipilosus* is closer to *T*. *piniperda* than *T*. *yunnanensis* and *T*.*minor*. *D*. *valens* is most closely related to *T*. *piniperda* in the genus *Tomicus* and *Hylurgus*, and most distantly related to *H*. *ligniperda*. Based on the analysis of the aforementioned results, it is possible to tentatively conclude that the Hylurgini beetles located in the same region are not closer to the genetic distance. Although their ecological niches are much closer, they have no obvious connection. However, since we only discussed a limited number of data of the Hylurgini, there is no evidence of other tribes, so further in-depth research is needed.

## 5. Conclusion

We analysed the structure and base composition, relative synonymous codon usage of protein-coding genes, secondary structure of tRNAs and synonymous substitution rate of the mitochondrial genomes of seven bark beetles. The conserved structure of the subfamily Scolytinae’s mitochondrial genome may help us elucidate the phylogenetic relationships at a relatively high taxonomic level. We constructed a phylogenetic tree based on its mitochondrial genome data. The phylogenetic analysis was conducted to understand the evolutionary relationships within the Hylurgini tribe and Scolytinae. The taxonomic position of Hylurgini is further clarified, as well as its relationship to other sister groups. The mitochondrial genome of Hylurgini will facilitate our search for more reliable regions to develop molecular markers, and not just the commonly used *COI* genes. The complex co-existence phenomenon of the Hylurgini is present not only in the same genus but also between different genera (native and non-native). This phenomenon complicates the identification of bark beetles further. The novel molecular markers will facilitate the challenging species identification of the Hylurgini.

## Supporting information

S1 Table The species information from GeneBank used in the article. (DOCX)

S2 Table Base composition of mitochondrial genomes of 37 species. (XLSX)

S3 Table Genetic distance of 37 species of beetles. (XLS)

S4 Table Organization of the mitochondrial genome of *Dendroctonus valens*. (DOCX)

S5 Table Organization of the mitochondrial genome of *Hylurgus ligniperda*. (DOCX)

S6 Table Organization of the mitochondrial genome of *Hylurgus micklitzi*. (DOCX)

S7 Table Organization of the mitochondrial genome of *Tomicus brevipilosus*. (DOCX)

S8 Table Organization of the mitochondrial genome of *Tomicus piniperda*. (DOCX)

S9 Table Organization of the mitochondrial genome of *Tomicus minor*. (DOCX)

S10 Table Organization of the mitochondrial genome of *Tomicus yunnanensis*. (DOCX)

S11 Table The data of dN, dS and dN/dS boxplot. (XLSX)

S12 Table The data of heatmap. (XLSX)

S13 Table World distribution of seven Hylurgini species. (DOCX)
