# Supplementary material for: Seven mitochondrial genomes of tribe Hylurgini (Coleoptera: Curculionidae: Scolytinae) in Eurasia and their phylogenetic analysis
Source: PLoS One. 2024 Nov 5;19(11):e0313448. doi: 10.1371/journal.pone.0313448 (PMC11537409; doi:10.1371/journal.pone.0313448)
Supplement: S1 Table — (DOCX) [file pone.0313448.s001.docx]

S1 Table. The species information from GeneBank used in the article.

| **Species** | **Accession** **no.** | **Species** | **Accession** **no.** |
| --- | --- | --- | --- |
| *Hylates* *brunneus* | KX035208.1 | *Dryocoetes autographus* | KX035207.1 |
| *Hylates* *attenuatus* | KX035212.1 | *Dryocoetes hectographus* | MZ766132.1 |
| *Hylastes brunneus* | KX035208.1 | *Dryocoetes villosus* | KX035216.1 |
| *Dendroctonus rufipennis* | MW487808.1 | *Trypodendron domesticum* | KX035205.1 |
| *Dendroctonus valens* | OL689184.1 | *Trypodendron signatum* | KX035214.1 |
| *Phloeosinus perlatus* | MW447510.1 | *Euwallacea fornicatus* | MT897842.1 |
| *Polygraphus poligraphus* | MN528600.1 | *Xyleborus dispar* | KX035217.1 |
| *Scolytus schevyrewi* | MK636870.1 | *Xylosandrus crassiusculus* | KX035196.1 |
| *Scolytus seulensis* | MK636869.1 | *Xylosandrus germanus* | KX035202.1 |
| *Ips acuminatus* | MK988441.1 | *Xylosandrus morigerus* | KX035191.1 |
| *Ips calligraphus* | MW589547.1 | *Coptodryas elegans* | OK539700.1 |
| *Ips sexdentatus* | KX035215.1 | *Trypophloeus asperatus* | KX035204.1 |
| *Orthotomicus erosus* | MZ823388.1 | *Pityophthorus pubescens* | KX035209.1 |
| *Orthotomicus laricis* | KX035213.1 | *Gnathotrichus materiarius* | KX035218.1 |
| *Pityogenes bidentatus* | KX035211.1 | *Cyclorhipidion bodoanus* | KX035219.1 |
