# Supplementary material for: Seven mitochondrial genomes of tribe Hylurgini (Coleoptera: Curculionidae: Scolytinae) in Eurasia and their phylogenetic analysis
Source: PLoS One. 2024 Nov 5;19(11):e0313448. doi: 10.1371/journal.pone.0313448 (PMC11537409; doi:10.1371/journal.pone.0313448)
Supplement: S4 Table — (DOCX) [file pone.0313448.s004.docx]

S4 Table. Organization of the mitochondrial genome of *Dendroctonus valens.*

| Gene | Majority(J)/minority(N) strand | Location | Size | Anticodon | Codon |  | Intergenic |
| --- | --- | --- | --- | --- | --- | --- | --- |
|  |  |  |  |  | Start | Stop | Nucleotides* |
| *tRNA^Gln^* | N | 1-69 | 69 | 37-39 TTG |  |  |  |
| *tRNA^Met^* | J | 76-145 | 70 | 106-108 CAT |  |  | 6 |
| *ND2* | J | 146-1162 | 1017 |  | ATT | TAA | 0 |
| *tRNA^Trp^* | J | 1161-1224 | 64 | 1191-1193 TCA |  |  | -2 |
| *tRNA^Cys^* | N | 1224-1283 | 60 | 1252-1254 GCA |  |  | -1 |
| *tRNA^Tyr^* | N | 1287-1348 | 62 | 1317-1319 GTA |  |  | 3 |
| *COI* | J | 1341-2885 | 1545 |  | ATT | TAA | -8 |
| *tRNA^Leu(UUR)^* | J | 2881-2945 | 65 | 2910-2912 TAA |  |  | -5 |
| *COII* | J | 2946-3629 | 684 |  | ATT | TAA | 0 |
| *tRNA^Lys^* | J | 3631-3701 | 71 | 3662-3664 CTT |  |  | 1 |
| *tRNA^Asp^* | J | 3701-3764 | 64 | 3731-3733 GTC |  |  | -1 |
| *ATP8* | J | 3765-3920 | 156 |  | ATC | TAA | 0 |
| *ATP6* | J | 3914-4585 | 672 |  | ATG | TAA | -7 |
| *COIII* | J | 4591-5373 | 783 |  | ATG | TAA | 5 |
| *tRNA^Gly^* | J | 5380-5442 | 63 | 5410-5412 TCC |  |  | 6 |
| *ND3* | J | 5443-5796 | 354 |  | ATT | TAG | 0 |
| *tRNA^Ala^* | J | 5795-5855 | 61 | 5824-5826 TGC |  |  | -2 |
| *tRNA^Arg^* | J | 5854-5921 | 68 | 5884-5886 TCG |  |  | -2 |
| *tRNA^Asn^* | J | 5920-5985 | 66 | 5950-5952 GTT |  |  | -2 |
| *tRNA^Ser(AGN)^* | J | 5986-6051 | 66 | 6011-6013 TCT |  |  | 0 |
| *tRNA^Glu^* | J | 6051-6115 | 65 | 6081-6083 TTC |  |  | -1 |
| *tRNA^Phe^* | N | 6114-6177 | 64 | 6143-6145 GAA |  |  | -2 |
| *ND5* | N | 6178-7873 | 1696 |  | ATT | T- | 0 |
| *tRNA^His^* | N | 7886-7946 | 61 | 7915-7917 GTG |  |  | 12 |
| *ND4* | N | 7947-9276 | 1330 |  | ATG | T- | 0 |
| *ND4L* | N | 9270-9560 | 291 |  | ATG | TAA | -7 |
| *tRNA^Thr^* | J | 9570-9632 | 63 | 9600-9602 TGT |  |  | 9 |
| *tRNA^Pro^* | N | 9633-9695 | 63 | 9664-9666 TGG |  |  | 0 |
| *ND6* | J | 9698-10198 | 501 |  | ATT | TAA | 2 |
| *Cytb* | J | 10198-11337 | 1140 |  | ATG | TAA | -1 |
| *tRNA^Ser(UCN)^* | J | 11341-11405 | 65 | 11370-11372 TGA |  |  | 3 |
| *ND1* | N | 11423-12352 | 930 |  | ATG | TAG | 17 |
| *tRNA^Leu(CUN)^* | N | 12372-12433 | 62 | 12402-12404 TAG |  |  | 19 |
| *lrRNA* | N | 12437-13735 | 1299 |  |  |  | 3 |
| *tRNA^Val^* | N | 13736-13802 | 67 | 13769-13771 TAC |  |  | 0 |
| *srRNA* | N | 13800-14569 | 770 |  |  |  | -3 |
| *tRNA^Ile^* | J | 14813-14877 | 65 | 14842-14844 GAT |  |  | 243 |
| *Control region* |  | 14878-16541 | 1664 |  |  |  | 0 |

* The number of nucleotides located between genes; negative numbers indicate that adjacent genes overlap.
