# Supplementary material for: Seven mitochondrial genomes of tribe Hylurgini (Coleoptera: Curculionidae: Scolytinae) in Eurasia and their phylogenetic analysis
Source: PLoS One. 2024 Nov 5;19(11):e0313448. doi: 10.1371/journal.pone.0313448 (PMC11537409; doi:10.1371/journal.pone.0313448)
Supplement: S5 Table — (DOCX) [file pone.0313448.s005.docx]

S5 Table. Organization of the mitochondrial genome of *Hylurgus ligniperda.*

| Gene | Majority(J)/minority(N) strand | Location | Size | Anticodon | Codon |  | Intergenic |
| --- | --- | --- | --- | --- | --- | --- | --- |
|  |  |  |  |  | Start | Stop | Nucleotides* |
| *tRNA^Gln^* | N | 1-69 | 69 | 37-39 TTG |  |  |  |
| *tRNA^Met^* | J | 76-145 | 70 | 107-109 CAT |  |  | 6 |
| *ND2* | J | 149-1156 | 1008 |  | ATT | TAA | 3 |
| *tRNA^Trp^* | J | 1155-1219 | 65 | 1186-1188 TCA |  |  | -2 |
| *tRNA^Cys^* | N | 1219-1282 | 64 | 1250-1252 GCA |  |  | -1 |
| *tRNA^Tyr^* | N | 1288-1352 | 65 | 1320-1322 GTA |  |  | 5 |
| *COI* | J | 1345-2892 | 1548 |  | ATC | TAA | -8 |
| *tRNA^Leu(UUR)^* | J | 2888-2952 | 65 | 2917-2919 TAA |  |  | -5 |
| *COII* | J | 2953-3639 | 687 |  | ATT | TAA | 0 |
| *tRNA^Lys^* | J | 3644-3711 | 68 | 3674-3676 CTT |  |  | 4 |
| *tRNA^Asp^* | J | 3711-3773 | 63 | 3741-3743 GTC |  |  | -1 |
| *ATP8* | J | 3774-3929 | 156 |  | ATT | TAA | 0 |
| *ATP6* | J | 3923-4600 | 678 |  | ATG | TAA | -7 |
| *COIII* | J | 4600-5382 | 783 |  | ATG | TAA | -1 |
| *tRNA^Gly^* | J | 5386-5451 | 66 | 5416-5418 TCC |  |  | 3 |
| *ND3* | J | 5452-5805 | 354 |  | ATA | TAA | 0 |
| *tRNA^Ala^* | J | 5805-5867 | 63 | 5834-5836 TGC |  |  | -1 |
| *tRNA^Arg^* | J | 5867-5933 | 67 | 5895-5897 TCG |  |  | -1 |
| *tRNA^Asn^* | J | 5932-5996 | 65 | 5962-5964 GTT |  |  | -2 |
| *tRNA^Ser(AGN)^* | J | 5997-6062 | 66 | 6022-6024 TCT |  |  | 0 |
| *tRNA^Glu^* | J | 6068-6133 | 66 | 6096-6098 TTC |  |  | 5 |
| *tRNA^Phe^* | N | 6134-6197 | 64 | 6164-6166 GAA |  |  | 0 |
| *ND5* | N | 6197-7908 | 1712 |  | ATT | TA- | -1 |
| *tRNA^His^* | N | 7909-7972 | 64 | 7940-7942 GTG |  |  | 0 |
| *ND4* | N | 7973-9299 | 1327 |  | ATG | T- | 0 |
| *ND4L* | N | 9303-9596 | 294 |  | ATG | TAG | 3 |
| *tRNA^Thr^* | J | 9605-9669 | 65 | 9635-9637 TGT |  |  | 8 |
| *tRNA^Pro^* | N | 9670-9735 | 66 | 9704-9706 TGG |  |  | 0 |
| *ND6* | J | 9738-10244 | 507 |  | ATT | TAA | 2 |
| *Cytb* | J | 10244-11383 | 1140 |  | ATG | TAA | -1 |
| *tRNA^Ser(UCN)^* | J | 11383-11451 | 69 | 11412-11414 TGA |  |  | -1 |
| *ND1* | N | 11469-12398 | 930 |  | ATA | TAA | 17 |
| *tRNA^Leu(CUN)^* | N | 12418-12481 | 64 | 12450-12452 TAG |  |  | 19 |
| *lrRNA* | N | 12485-13777 | 1293 |  |  |  | 3 |
| *tRNA^Val^* | N | 13778-13843 | 66 | 13811-13813 TAC |  |  | 0 |
| *srRNA* | N | 13842-14611 | 770 |  |  |  | -2 |
| *Control region* |  | 14612-16106 | 1495 |  |  |  | 0 |

* The number of nucleotides located between genes; negative numbers indicate that adjacent genes overlap.
