# Supplementary material for: Seven mitochondrial genomes of tribe Hylurgini (Coleoptera: Curculionidae: Scolytinae) in Eurasia and their phylogenetic analysis
Source: PLoS One. 2024 Nov 5;19(11):e0313448. doi: 10.1371/journal.pone.0313448 (PMC11537409; doi:10.1371/journal.pone.0313448)
Supplement: S6 Table — (DOCX) [file pone.0313448.s006.docx]

S6 Table. Organization of the mitochondrial genome of *Hylurgus micklitzi.*

| Gene | Majority(J)/minority(N) strand | Location | Size | Anticodon | Codon |  | Intergenic |
| --- | --- | --- | --- | --- | --- | --- | --- |
|  |  |  |  |  | Start | Stop | Nucleotides* |
| *tRNA^Gln^* | N | 1-69 | 69 | 37-39 TTG |  |  |  |
| *tRNA^Met^* | J | 73-140 | 68 | 104-106 CAT |  |  | 3 |
| *ND2* | J | 144-1154 | 1011 |  | ATT | TAA | 3 |
| *tRNA^Trp^* | J | 1153-1215 | 63 | 1183-1185 TCA |  |  | -2 |
| *tRNA^Cys^* | N | 1215-1283 | 69 | 1252-1254 GCA |  |  | -1 |
| *tRNA^Tyr^* | N | 1286-1347 | 62 | 1316-1318 GTA |  |  | 2 |
| *COI* | J | 1340-2884 | 1545 |  | ATT | TAA | -8 |
| *tRNA^Leu(UUR)^* | J | 2880-2944 | 65 | 2909-2911 TAA |  |  | -5 |
| *COII* | J | 2945-3628 | 684 |  | ATC | TAA | 0 |
| *tRNA^Lys^* | J | 3634-3704 | 71 | 3664-3666 CTT |  |  | 5 |
| *tRNA^Asp^* | J | 3704-3765 | 62 | 3734-3736 GTC |  |  | -1 |
| *ATP8* | J | 3766-3921 | 156 |  | ATT | TAA | 0 |
| *ATP6* | J | 3915-4592 | 678 |  | ATG | TAA | -7 |
| *COIII* | J | 4592-5374 | 783 |  | ATG | TAA | -1 |
| *tRNA^Gly^* | J | 5388-5452 | 65 | 5420-5422 TCC |  |  | 13 |
| *ND3* | J | 5453-5806 | 354 |  | ATA | TAG | 0 |
| *tRNA^Ala^* | J | 5805-5867 | 63 | 5833-5835 TGC |  |  | -2 |
| *tRNA^Arg^* | J | 5871-5935 | 65 | 5899-5901 TCG |  |  | 3 |
| *tRNA^Asn^* | J | 5934-5996 | 63 | 5964-5966 GTT |  |  | -2 |
| *tRNA^Ser(AGN)^* | J | 5997-6062 | 66 | 6022-6024 TCT |  |  | 0 |
| *tRNA^Glu^* | J | 6063-6127 | 65 | 6091-6093 TTC |  |  | 0 |
| *tRNA^Phe^* | N | 6126-6187 | 62 | 6155-6157 GAA |  |  | -2 |
| *ND5* | N | 6188-7856 | 1669 |  | ATT | T- | 0 |
| *tRNA^His^* | N | 7902-7967 | 66 | 7933-7935 GTG |  |  | 45 |
| *ND4* | N | 7968-9297 | 1330 |  | ATG | T- | 0 |
| *ND4L* | N | 9299-9592 | 294 |  | ATG | TAA | 1 |
| *tRNA^Thr^* | J | 9596-9658 | 63 | 9626-9628 TGT |  |  | 3 |
| *tRNA^Pro^* | N | 9659-9722 | 64 | 9691-9693 TGG |  |  | 0 |
| *ND6* | J | 9731-10234 | 504 |  | ATT | TAA | 8 |
| *Cytb* | J | 10234-11373 | 1140 |  | ATG | TAG | -1 |
| *tRNA^Ser(UCN)^* | J | 11372-11437 | 66 | 11401-11403 TGA |  |  | -2 |
| *ND1* | N | 11455-12402 | 948 |  | TTG | TAA | 17 |
| *tRNA^Leu(CUN)^* | N | 12404-12466 | 63 | 12435-12437 TAG |  |  | 1 |
| *lrRNA* | N | 12467-13749 | 1283 |  |  |  | 0 |
| *tRNA^Val^* | N | 13750-13815 | 66 | 13783-13785 TAC |  |  | 0 |
| *srRNA* | N | 13816-14580 | 765 |  |  |  | 0 |
| *Control region* |  | 14581-15378 | 798 |  |  |  | 0 |

* The number of nucleotides located between genes; negative numbers indicate that adjacent genes overlap.
