# Supplementary material for: Seven mitochondrial genomes of tribe Hylurgini (Coleoptera: Curculionidae: Scolytinae) in Eurasia and their phylogenetic analysis
Source: PLoS One. 2024 Nov 5;19(11):e0313448. doi: 10.1371/journal.pone.0313448 (PMC11537409; doi:10.1371/journal.pone.0313448)
Supplement: S7 Table — (DOCX) [file pone.0313448.s007.docx]

S7 Table. Organization of the mitochondrial genome of *Tomicus brevipilosus.*

| Gene | Majority(J)/minority(N) strand | Location | Size | Anticodon | Codon |  | Intergenic |
| --- | --- | --- | --- | --- | --- | --- | --- |
|  |  |  |  |  | Start | Stop | Nucleotides* |
| *tRNA^Gln^* | N | 1-69 | 69 | 37-39 TTG |  |  |  |
| *tRNA^Met^* | J | 87-155 | 69 | 117-119 CAT |  |  | 7 |
| *ND2* | J | 177-1175 | 999 |  | ATT | TAA | 21 |
| *tRNA^Trp^* | J | 1184-1249 | 66 | 1214-1216 TCA |  |  | 8 |
| *tRNA^Cys^* | N | 1258-1325 | 68 | 1294-1296 GCA |  |  | 8 |
| *tRNA^Tyr^* | N | 1353-1414 | 62 | 1383-1385 GTA |  |  | 27 |
| *COI* | J | 1407-2948 | 1542 |  | ATT | TAA | -8 |
| *tRNA^Leu(UUR)^* | J | 2960-3024 | 65 | 2989-2991 TAA |  |  | 1 |
| *COII* | J | 3025-3706 | 682 |  | ATT | T- | 0 |
| *tRNA^Lys^* | J | 3707-3777 | 71 | 3737-3739 CTT |  |  | 0 |
| *tRNA^Asp^* | J | 3777-3839 | 63 | 3807-3809 GTC |  |  | -1 |
| *ATP8* | J | 3840-3998 | 159 |  | ATT | TAA | 0 |
| *ATP6* | J | 3992-4666 | 675 |  | ATG | TAA | -7 |
| *COIII* | J | 4666-5448 | 783 |  | ATG | TAA | -1 |
| *tRNA^Gly^* | J | 5473-5538 | 66 | 5504-5506 TCC |  |  | 24 |
| *ND3* | J | 5539-5892 | 354 |  | ATT | TAA | 0 |
| *tRNA^Ala^* | J | 5900-5965 | 66 | 5928-5930 TGC |  |  | 7 |
| *tRNA^Arg^* | J | 5965-6029 | 65 | 5993-5995 TCG |  |  | -1 |
| *tRNA^Asn^* | J | 6032-6095 | 64 | 6062-6064 GTT |  |  | 2 |
| *tRNA^Ser(AGN)^* | J | 6096-6162 | 67 | 6121-6123 TCT |  |  | 0 |
| *tRNA^Glu^* | J | 6164-6228 | 65 | 6192-6194 TTC |  |  | 1 |
| *tRNA^Phe^* | N | 6240-6304 | 65 | 6269-6271 GAA |  |  | 11 |
| *ND5* | N | 6301-8023 | 1732 |  | ATA | T- | 4 |
| *tRNA^His^* | N | 8024-8087 | 64 | 8054-8056 GTG |  |  | 0 |
| *ND4* | N | 8088-9420 | 1333 |  | ATG | T- | 0 |
| *ND4L* | N | 9414-9707 | 294 |  | ATG | TAA | -7 |
| *tRNA^Thr^* | J | 9710-9774 | 65 | 9740-9742 TGT |  |  | 2 |
| *tRNA^Pro^* | N | 9775-9837 | 63 | 9806-9808 TGG |  |  | 0 |
| *ND6* | J | 9849-10349 | 501 |  | ATT | TAA | 11 |
| *Cytb* | J | 10349-11488 | 1140 |  | ATG | TAG | -1 |
| *tRNA^Ser(UCN)^* | J | 11487-11553 | 67 | 11516-11518 TGA |  |  | -2 |
| *ND1* | N | 11571-12515 | 945 |  | TTG | TAA | 17 |
| *tRNA^Leu(CUN)^* | N | 12520-12582 | 63 | 12551-12553 TAG |  |  | 4 |
| *lrRNA* | N | 12583-13874 | 1292 |  |  |  | 0 |
| *tRNA^Val^* | N | 13875-13940 | 66 | 13908-13910 TAC |  |  | 0 |
| *srRNA* | N | 13940-14717 | 778 |  |  |  | -1 |
| *Control region* |  | 14718-16390 | 1673 |  |  |  | 0 |

* The number of nucleotides located between genes; negative numbers indicate that adjacent genes overlap.
