# Supplementary material for: Seven mitochondrial genomes of tribe Hylurgini (Coleoptera: Curculionidae: Scolytinae) in Eurasia and their phylogenetic analysis
Source: PLoS One. 2024 Nov 5;19(11):e0313448. doi: 10.1371/journal.pone.0313448 (PMC11537409; doi:10.1371/journal.pone.0313448)
Supplement: S8 Table — (DOCX) [file pone.0313448.s008.docx]

S8 Table. Organization of the mitochondrial genome of *Tomicus piniperda.*

| Gene | Majority(J)/minority(N) strand | Location | Size | Anticodon | Codon |  | Intergenic |
| --- | --- | --- | --- | --- | --- | --- | --- |
|  |  |  |  |  | Start | Stop | Nucleotides* |
| *tRNA^Gln^* | N | 1-69 | 69 | 37-39 TTG |  |  |  |
| *tRNA^Met^* | J | 76-144 | 69 | 106-108 CAT |  |  | 6 |
| *ND2* | J | 145-1164 | 1020 |  | ATT | TAA | 0 |
| *tRNA^Trp^* | J | 1167-1233 | 67 | 1197-1199 TCA |  |  | 2 |
| *tRNA^Cys^* | N | 1267-1333 | 67 | 1302-1304 GCA |  |  | 33 |
| *tRNA^Tyr^* | N | 1336-1398 | 63 | 1367-1369 GTA |  |  | 2 |
| *COI* | J | 1391-2932 | 1542 |  | ATT | TAA | -8 |
| *tRNA^Leu(UUR)^* | J | 2937-3001 | 65 | 2966-2968 TAA |  |  | 4 |
| *COII* | J | 3002-3683 | 682 |  | ATT | T- | 0 |
| *tRNA^Lys^* | J | 3684-3754 | 71 | 3714-3716 CTT |  |  | 0 |
| *tRNA^Asp^* | J | 3754-3816 | 63 | 3784-3786 GTC |  |  | -1 |
| *ATP8* | J | 3817-3975 | 159 |  | ATT | TAA | 0 |
| *ATP6* | J | 3969-4643 | 675 |  | ATG | TAA | -7 |
| *COIII* | J | 4643-5425 | 783 |  | ATG | TAA | -1 |
| *tRNA^Gly^* | J | 5438-5503 | 66 | 5469-5471 TCC |  |  | 12 |
| *ND3* | J | 5504-5857 | 354 |  | ATT | TAA | 0 |
| *tRNA^Ala^* | J | 5865-5932 | 68 | 5894-5896 TGC |  |  | 7 |
| *tRNA^Arg^* | J | 5932-5995 | 64 | 5960-5962 TCG |  |  | -1 |
| *tRNA^Asn^* | J | 5996-6059 | 64 | 6026-6028 GTT |  |  | 0 |
| *tRNA^Ser(AGN)^* | J | 6060-6126 | 67 | 6085-6087 TCT |  |  | 0 |
| *tRNA^Glu^* | J | 6128-6190 | 63 | 6158-6160 TTC |  |  | 1 |
| *tRNA^Phe^* | N | 6189-6253 | 65 | 6219-6221 GAA |  |  | -2 |
| *ND5* | N | 6261-7979 | 1719 |  | ATT | TAG | 7 |
| *tRNA^His^* | N | 7983-8045 | 63 | 8014-8016 GTG |  |  | 3 |
| *ND4* | N | 8046-9378 | 1333 |  | ATG | T- | 0 |
| *ND4L* | N | 9372-9659 | 288 |  | ATA | TAA | -7 |
| *tRNA^Thr^* | J | 9668-9731 | 64 | 9698-9700 TGT |  |  | 8 |
| *tRNA^Pro^* | N | 9732-9794 | 63 | 9763-9765 TGG |  |  | 0 |
| *ND6* | J | 9806-10300 | 495 |  | ATT | TAA | 11 |
| *Cytb* | J | 10303-11442 | 1140 |  | ATG | TAA | 2 |
| *tRNA^Ser(UCN)^* | J | 11447-11513 | 67 | 11476-11478 TGA |  |  | 4 |
| *ND1* | N | 11531-12478 | 948 |  | TTG | TAA | 17 |
| *tRNA^Leu(CUN)^* | N | 12480-12542 | 63 | 12511-12513 TAG |  |  | 1 |
| *lrRNA* | N | 12543-13839 | 1297 |  |  |  | 0 |
| *tRNA^Val^* | N | 13840-13904 | 65 | 13873-13875 TAC |  |  | 0 |
| *srRNA* | N | 13904-14680 | 777 |  |  |  | -1 |
| *Control region* |  | 14681-15339 | 659 |  |  |  | 0 |

* The number of nucleotides located between genes; negative numbers indicate that adjacent genes overlap.
