# Supplementary material for: Seven mitochondrial genomes of tribe Hylurgini (Coleoptera: Curculionidae: Scolytinae) in Eurasia and their phylogenetic analysis
Source: PLoS One. 2024 Nov 5;19(11):e0313448. doi: 10.1371/journal.pone.0313448 (PMC11537409; doi:10.1371/journal.pone.0313448)
Supplement: S9 Table — (DOCX) [file pone.0313448.s009.docx]

S9 Table. Organization of the mitochondrial genome of *Tomicus minor.*

| Gene | Majority(J)/minority(N) strand | Location | Size | Anticodon | Codon |  | Intergenic |
| --- | --- | --- | --- | --- | --- | --- | --- |
|  |  |  |  |  | Start | Stop | Nucleotides* |
| *tRNA^Ile^* | J | 17955-65 | 67 | 28-30 GAT |  |  |  |
| *tRNA^Gln^* | N | 94-161 | 68 | 130-132 TTG |  |  | 28 |
| *tRNA^Met^* | J | 161-228 | 68 | 191-193 CAT |  |  | -1 |
| *ND2* | J | 250-1248 | 999 |  | ATT | TAA | 21 |
| *tRNA^Trp^* | J | 1249-1317 | 69 | 1281-1283 TCA |  |  | 0 |
| *tRNA^Cys^* | N | 1462-1527 | 66 | 1494-1496 GCA |  |  | 144 |
| *tRNA^Tyr^* | N | 1574-1636 | 63 | 1605-1607 GTA |  |  | 46 |
| *COI* | J | 1639-3174 | 1536 |  | TCG | TAA | 2 |
| *tRNA^Leu(UUR)^* | J | 3178-3242 | 65 | 3207-3209 TAA |  |  | 3 |
| *COII* | J | 3243-3924 | 682 |  | ATC | T- | 0 |
| *tRNA^Lys^* | J | 3925-3995 | 71 | 3955-3957 CTT |  |  | 0 |
| *tRNA^Asp^* | J | 3995-4058 | 64 | 4025-4027 GTC |  |  | -1 |
| *ATP8* | J | 4059-4217 | 159 |  | ATT | TAG | 0 |
| *ATP6* | J | 4211-4885 | 675 |  | ATG | TAA | -7 |
| *COIII* | J | 4885-5667 | 783 |  | ATG | TAA | -1 |
| *tRNA^Gly^* | J | 5695-5761 | 67 | 5726-5728 TCC |  |  | 27 |
| *ND3* | J | 5762-6115 | 354 |  | ATA | TAG | 0 |
| *tRNA^Ala^* | J | 6121-6187 | 67 | 6150-6152 TGC |  |  | 5 |
| *tRNA^Arg^* | J | 6187-6225 | 69 | 6215-6217 TCG |  |  | -1 |
| *tRNA^Asn^* | J | 6256-6327 | 72 | 6286-6288 GTT |  |  | 30 |
| *tRNA^Ser(AGN)^* | J | 6328-6394 | 67 | 6353-6355 TCT |  |  | 0 |
| *tRNA^Glu^* | J | 6396-6457 | 62 | 6426-6428 TTC |  |  | 1 |
| *tRNA^Phe^* | N | 6456-6521 | 66 | 6489-6491 GAA |  |  | -2 |
| *ND5* | N | 6522-8235 | 1714 |  | ATT | T- | 0 |
| *tRNA^His^* | N | 8242-8305 | 64 | 8274-8276 GTG |  |  | 6 |
| *ND4* | N | 8306-9638 | 1333 |  | ATG | T- | 0 |
| *ND4L* | N | 9632-9925 | 294 |  | ATG | TAA | -7 |
| *tRNA^Thr^* | J | 9929-9994 | 66 | 9960-9962 TGT |  |  | 3 |
| *tRNA^Pro^* | N | 9995-10056 | 62 | 10025-10027 TGG |  |  | 0 |
| *ND6* | J | 10059-10562 | 504 |  | ATT | TAA | 2 |
| *Cytb* | J | 10564-11703 | 1140 |  | ATG | TAA | 1 |
| *tRNA^Ser(UCN)^* | J | 11704-11771 | 68 | 11733-11735 TGA |  |  | 0 |
| *ND1* | N | 11892-12824 | 933 |  | ATT | TAA | 120 |
| *tRNA^Leu(CUN)^* | N | 12841-12903 | 63 | 12872-12874 TAG |  |  | 16 |
| *lrRNA* | N | 12904-14200 | 1297 |  |  |  | 0 |
| *tRNA^Val^* | N | 14201-14266 | 66 | 14234-14236 TAC |  |  | 0 |
| *srRNA* | N | 14266-15047 | 782 |  |  |  | -1 |
| *Control region* |  | 15048-17954 | 2907 |  |  |  | 0 |

* The number of nucleotides located between genes; negative numbers indicate that adjacent genes overlap.
