# Supplementary material for: Seven mitochondrial genomes of tribe Hylurgini (Coleoptera: Curculionidae: Scolytinae) in Eurasia and their phylogenetic analysis
Source: PLoS One. 2024 Nov 5;19(11):e0313448. doi: 10.1371/journal.pone.0313448 (PMC11537409; doi:10.1371/journal.pone.0313448)
Supplement: S10 Table — (DOCX) [file pone.0313448.s010.docx]

S10 Table. Organization of the mitochondrial genome of *Tomicus yunnanensis.*

| Gene | Majority(J)/minority(N) strand | Location | Size | Anticodon | Codon |  | Intergenic Nucleotides* |
| --- | --- | --- | --- | --- | --- | --- | --- |
|  |  |  |  |  | Start | Stop |  |
| *tRNA^Ile^* | J | 1-65 | 65 | 30-32 GAT |  |  |  |
| *tRNA^Gln^* | N | 346-414 | 69 | 382-384 TTG |  |  | 281 |
| *tRNA^Met^* | J | 428-496 | 69 | 458-460 CAT |  |  | 13 |
| *ND2* | J | 518-1516 | 999 |  | ATT | TAA | 21 |
| *tRNA^Trp^* | J | 1515-1580 | 66 | 1546-1548 TCA |  |  | -2 |
| *tRNA^Cys^* | N | 1635-1699 | 65 | 1668-1670 GCA |  |  | 54 |
| *tRNA^Tyr^* | N | 1726-1791 | 66 | 1758-1760 GTA |  |  | 26 |
| *COI* | J | 1784-3325 | 1542 |  | ATT | TAA | -8 |
| *tRNA^Leu(UUR)^* | J | 3330-3395 | 66 | 3360-3362 TAA |  |  | 4 |
| *COII* | J | 3396-4077 | 682 |  | ATT | T- | 0 |
| *tRNA^Lys^* | J | 4078-4148 | 71 | 4108-4110 CTT |  |  | 0 |
| *tRNA^Asp^* | J | 4148-4211 | 64 | 4178-4180 GTC |  |  | -1 |
| *ATP8* | J | 4212-4370 | 159 |  | ATT | TAA | 0 |
| *ATP6* | J | 4364-5038 | 675 |  | ATG | TAA | -7 |
| *COIII* | J | 5038-5820 | 783 |  | ATG | TAA | -1 |
| *tRNA^Gly^* | J | 5841-5906 | 66 | 5872-5874 TCC |  |  | 20 |
| *ND3* | J | 5907-6260 | 354 |  | ATT | TAA | 0 |
| *tRNA^Ala^* | J | 6273-6337 | 65 | 6301-6303 TGC |  |  | 12 |
| *tRNA^Arg^* | J | 6337-6400 | 64 | 6365-6367 TCG |  |  | -1 |
| *tRNA^Asn^* | J | 6402-6465 | 64 | 6432-6434 GTT |  |  | 1 |
| *tRNA^Ser(AGN)^* | J | 6466-6533 | 68 | 6491-6493 TCT |  |  | 0 |
| *tRNA^Glu^* | J | 6535-6597 | 63 | 6565-6567 TTC |  |  | 1 |
| *tRNA^Phe^* | N | 6596-6660 | 65 | 6626-6628 GAA |  |  | -2 |
| *ND5* | N | 6661-8332 | 1672 |  | ATT | T- | 0 |
| *tRNA^His^* | N | 8381-8444 | 64 | 8412-8414 GTG |  |  | 48 |
| *ND4* | N | 8445-9777 | 1333 |  | ATG | T- | 0 |
| *ND4L* | N | 9771-10064 | 294 |  | ATG | TAA | -7 |
| *tRNA^Thr^* | J | 10067-10129 | 63 | 10097-10099 TGT |  |  | 2 |
| *tRNA^Pro^* | N | 10130-10192 | 63 | 10161-10163 TGG |  |  | 0 |
| *ND6* | J | 10195-10698 | 504 |  | ATT | TAA | 2 |
| *Cytb* | J | 10700-11839 | 1140 |  | ATG | TAA | 1 |
| *tRNA^Ser(UCN)^* | J | 11848-11914 | 67 | 11877-11879 TGA |  |  | 8 |
| *ND1* | N | 11932-12876 | 945 |  | TTG | TAG | 17 |
| *tRNA^Leu(CUN)^* | N | 12881-12944 | 64 | 12913-12915 TAG |  |  | 4 |
| *lrRNA* | N | 12945-14245 | 1301 |  |  |  | 0 |
| *tRNA^Val^* | N | 14246-14311 | 66 | 14279-14281 TAC |  |  | 0 |
| *srRNA* | N | 14311-15085 | 775 |  |  |  | -1 |
| *Control region* |  | 15086-16088 | 1003 |  |  |  | 0 |

* The number of nucleotides located between genes; negative numbers indicate that adjacent genes overlap.
