# Supplementary material for: Seven mitochondrial genomes of tribe Hylurgini (Coleoptera: Curculionidae: Scolytinae) in Eurasia and their phylogenetic analysis
Source: PLoS One. 2024 Nov 5;19(11):e0313448. doi: 10.1371/journal.pone.0313448 (PMC11537409; doi:10.1371/journal.pone.0313448)
Supplement: S13 Table — (DOCX) [file pone.0313448.s013.docx]

**S13 Table. World distribution of seven Hylurgini species.**

| **Species** | **Country** | **Location** |
| --- | --- | --- |
| *Dendroctonus valens* | USA |  |
|  | Canada | southern Canada |
|  | Mexico |  |
|  | Honduras |  |
|  | China | **Hebei Province** (North latitude 36°05 '~ 42°40', East longitude 113°27 '~ 119°50') |
|  |  | **Henan** **Province** (North latitude 31°23 '~ 36°22', East longitude 110°21 '~ 116°39') |
|  |  | **Shanxi** **Province** (North latitude 34°34 '~40 °44', East longitude 110°14 '~114 °33',) |
| *Hylurgus ligniperda* | Ukraine | Crimea |
|  | Algeria |  |
|  | Türkiye | Asia Minor |
|  | Russia |  |
|  | Azerbaijan |  |
|  | Armenia |  |
|  | Georgia |  |
|  | Japan |  |
|  | Sri Lanka |  |
|  | Australia |  |
|  | New Zealand |  |
|  | USA | New York |
|  | Brazil |  |
|  | Chile |  |
|  | China | **Yantai City, Shandong Province** (North latitude 37°4', East longitude 121°85') |
| *Hylurgus micklitzi* | Spain |  |
|  | France | Le Thoronet (North latitude 43°45', East longitude 6°3') |
|  | Germany |  |
|  | Italy |  |
|  | Greece |  |
|  | Croatia |  |
|  | Hungary |  |
| *Tomicus piniperda* | Portugal | * Widely distributed in Asia and Europe |
|  | Japan |  |
|  | USA |  |
|  | Canada |  |
|  | China | **Inner Mongolia** (North latitude 41°4', East longitude 118°46') |
|  |  | **Yantai City, Shandong Province** (North latitude 37°4', East longitude 121°85') |
| *Tomicus brevipilosus* | India |  |
|  | Japan |  |
|  | Korea |  |
|  | Philippines |  |
|  | China | **Yunnan Province** (North latitude 25°3', East longitude 100°9') |
| *Tomicus minor* | Japan | * Widely distributed in Asia and Europe |
|  | Spain |  |
|  | France |  |
|  | Finland |  |
|  | Norway |  |
|  | Sweden |  |
|  | Russia |  |
|  | China | **Yunnan Province** (North latitude 25°3', East longitude 100°9') |
| *Tomicus yunnanensis* | China | **Yunnan Province** (North latitude 25°3', East longitude 100°9') |
|  |  | **Sichuan Province** (North latitude 26°05'~ 27°21', East longitude 101°08 '~ 102°15') |
